# Lactation undernutrition leads to multigenerational molecular programming of hypothalamic gene networks controlling reproduction

**DOI:** 10.1186/s12864-016-2615-4

**Published:** 2016-05-04

**Authors:** Monika M. Kaczmarek, Tamra Mendoza, Leslie P. Kozak

**Affiliations:** Institute of Animal Reproduction and Food Research, Polish Academy of Sciences, Olsztyn, Poland; Pennington Biomedical Research Center, Baton Rouge, Louisiana USA

**Keywords:** Hypothalamus, Kisspeptin, Leptin, Reproductive performance, Multigenerational programming

## Abstract

**Background:**

Reproductive success is dependent on development of hypothalamic circuits involving many hormonal systems working in concert to regulate gonadal function and sexual behavior. The timing of pubertal initiation and progression in mammals is likely influenced by the nutritional and metabolic state, leading us to the hypothesis that transient malnutrition experienced at critical times during development may perturb pubertal progression through successive generations. To test this hypothesis we have utilized a mouse model of undernutrition during suckling by exposing lactating mothers to undernutrition.

**Results:**

Using a combination of transcriptomic and biological approaches, we demonstrate that molecular programming of hypothalamus may perturb gender specific phenotypes across generations that are dependent on the nutritional environment of the lactation period. Lactation undernutrition in first (F1) generation offspring affected body composition, reproductive performance parameters and influenced the expression of genes responsible for hypothalamic neural circuits controlling reproductive function of both sexes. Strikingly, F2 offspring showed phenotypes similar to F1 progeny; however, they were sex and parental nutritional history specific. Here, we showed that deregulated expression of genes involved in kisspeptin signaling within the hypothalamus is strongly associated with a delay in the attainment of puberty in F1 and F2 male and female offspring.

**Conclusion:**

The early developmental plasticity of hypothalamus when challenged with undernutrition during postnatal development not only leads to altered expression of genes controlling hypothalamic neural circuits, altered body composition, delayed puberty and disturbed reproductive performance in F1 progeny, but also affects F2 offspring, depending on parental malnutrition history and in sexually dimorphic manner.

**Electronic supplementary material:**

The online version of this article (doi:10.1186/s12864-016-2615-4) contains supplementary material, which is available to authorized users.

## Background

Reproduction is an indispensable function for the preservation of the species and thus is under the control of a complex network of regulatory signals. While different reproductive strategies have been developed during evolution, in mammals and other species the regulatory factors mainly originate and/or integrate at the hypothalamic-pituitary-gonadal (HPG) axis. In general, the function of this neuro-hormonal axis relies primarily on the dynamic interaction of three major groups of signals arising from: 1) the hypothalamus, where a small population of scattered neurons synthesize and release gonadotropin-releasing hormone (GnRH); 2) the anterior pituitary, where gonadotropes secrete the gonadotropins - luteinizing hormone (LH), and follicle-stimulating hormone (FSH); and finally 3) the gonads that, in addition to producing gametes, are responsible for the synthesis and release of sex steroid and peptide hormones [1, 2].

Besides its dynamic regulation in adulthood (e.g. cyclical changes in menstrual/estrous cycle), the HPG axis undergoes significant maturation and functional changes during fetal and postnatal development [3], which particularly include sexual differentiation of the brain and the attainment of reproductive capacity at puberty. Importantly, the above mentioned phenomena display sexual dimorphism, and substantial differences are detected between males and females in relation to the development of reproductive brain circuits, the timing of puberty, and the function of the HPG axis in adulthood [1, 2]. In this circuitry, the essential contribution of several neuropeptide pathways, including prominently kisspeptin neurons, has been documented [4]. Kisspeptin neurons are widely accepted as conduits for transmitting metabolic information onto the centers governing the reproductive axis [5]. In this context, the ability of high doses of leptin to increase the hypothalamic expression of *Kiss1* gene in different models of severe metabolic stress, such as the leptin-deficient ob/ob mouse and the diabetic rat, fueled the hypothesis that leptin acts on kisspeptin neurons to conduct its stimulatory/permissive effects on GnRH neurons [6–8].

Metabolic conditions and the amount of body fuel reserves play a key role in the attainment of puberty [9, 10]. Thus, the attainment of reproductive capacity is only possible if proper fuel stores and metabolic conditions are acquired. Moreover, research in humans and a variety of animal models showed a link between early life nutrition and optimal reproductive function [10–12]. Undernutrition caused by anorexia or calorie restriction delays the onset of puberty in humans, disrupts estrous cycles, and delays the postpartum return to estrus in sexually mature animals. On the other hand, obese individuals share a phenotype similar to that of calorie-restricted individuals, i.e. a variety of alterations in the reproductive axis, including decreased fertility [13]. However, in contrast to undernutrition, which causes delayed puberty, childhood obesity induces precocious puberty in girls [14].

**Fig. 1:**
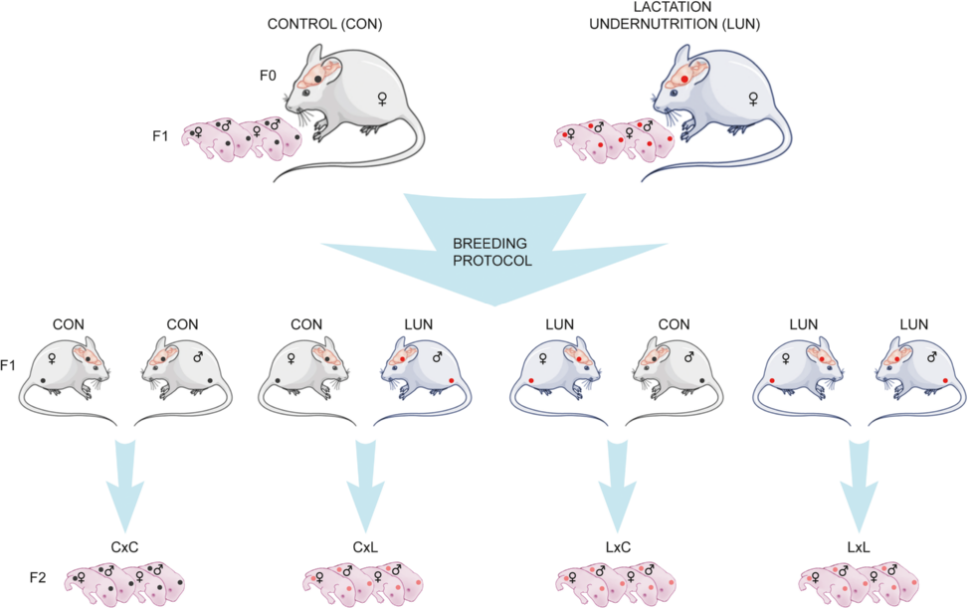
Nutritional and breeding strategy to generate the first- and second-generation offspring. Newborn F1 mice were raised from birth to weaning under control conditions (CON; F0 mother fed *ad libitum*) or lactation undernutrition condition (LUN; F0 mother fed 50 % of the food consumed by the control F0 mice) (upper panel). After weaning the F1 offspring from the all nutritional conditions were fed *ad libitum*. To generate the second-generation offspring (F2; lower panel), unrelated nonsibling F1 CON (C) and LUN (L) females (♀) and males (♂) were mated at age of 2 months in four combinations: C♀ x C♂, C♀ x L♂, L♀ x C♂, and L♀ x L♂ (middle panel). Lactating F1 females were not subjected to food restriction. After weaning the F2 offspring were fed *ad libitum*. Red dots represent possible sites of transcriptomic and functional modifications of HPG axis related reproductive circuits. Black dots – hypothalami and gonads of control animals

Evidence from human and experimental studies suggests that the timing of puberty can be affected by early metabolic influences taking place pre- or postnatally [15–18]. Although early life nutrition appears to be a critical requirement for optimal mammalian reproduction, the precise neurobiological mechanisms that contribute to this phenomenon are poorly understood. Thus in the present study we hypothesized that transient undernutrition experienced at critical times during development affects the development of hypothalamic gene networks involved in the HPG circuitry as well as subsequent reproductive performance. To address this hypothesis we have used a well-established mouse model of undernutrition during lactation causing severe leptin and insulin deficiency (Fig. 1) [19], to show that neonates exposed to undernutrition during lactation acquire sex-specific delays in the realization of puberty, possibly through molecular programming of the hypothalamus. Since kisspeptin neural circuits develop gradually during the first 2–5 weeks of postnatal life [20], when leptin and insulin deficiency occurs in our model, we examined hypothalamic gene networks controlling reproduction in 10- and 21-day old mice. Males and females with changed hypothalamic gene networks controlling reproduction showed altered body composition, delayed puberty and disturbed reproductive performance. Furthermore, our studies suggest that the molecular and physiological effects of postnatal undernutrition are multigenerational.

## Results

### Growth and body composition after undernutrition during early postnatal development

**Fig. 2:**
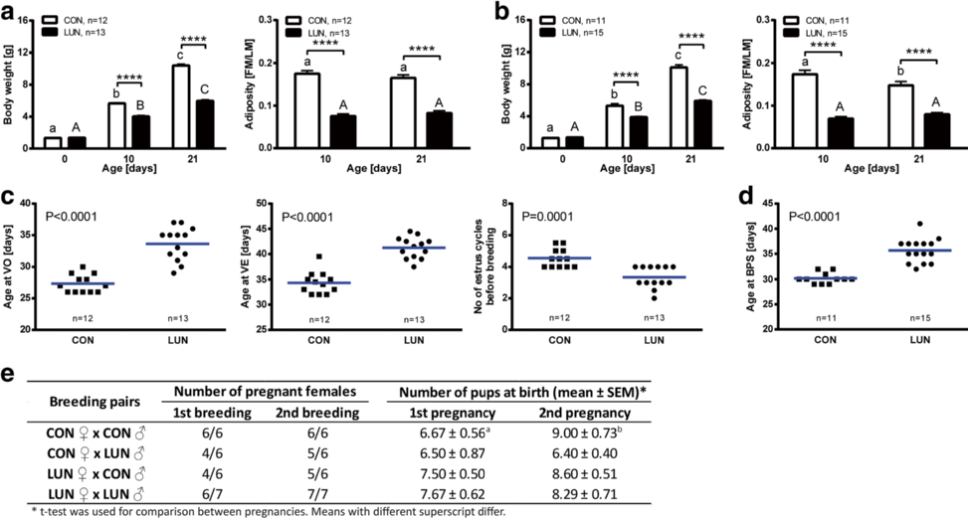
Effects of undernutrition (LUN) from birth to weaning at 21 days of age on body composition, puberty attainment, pregnancy rates and litter sizes in F1 progeny. Body weight and adiposity index (fat mass/lean mass [FM/LM]) in female (**a**) and male (**b**) progeny. Data are expressed as mean ± SEM. Means with different superscripts differ significantly (small letters – control (CON), capital letters – LUN). Asterisks indicate difference between nutritional protocols for the same day of age (*Two-way ANOVA*; ****, *P* < 0.0001). Timing of vaginal opening (VO; **c**), vaginal estrus (VE; **c**), number of estrus before breeding (at 55 days of age; **c**), and timing of balano preputial separation (BPS; **d**) in control (CON) and LUN mice (statistical significance was calculated by *t test*). Number of pregnant females and litter parameters in first and second pregnancy (**e**) for each breeding pair (CON ♀ x CON ♂, CON ♀ x LUN ♂, LUN ♀ x CON ♂, LUN ♀ x LUN ♂)

The design of the nutritional experiment, as described by Kozak and coworkers [19], aims to perturb body composition and hormonal homeostasis to assess attainment of puberty and patterns of gene expression in the hypothalamus of control mice (CON) and mice with undernutrition (LUN) during lactation (birth to 3 weeks of age). Neonatal undernutrition was associated with changes in growth as revealed by a significant decrease in the pre-weaning body weight of underfed compared with normal fed mice. As early as on day 10, LUN females (Fig. 2a) and males (Fig. 2b) had reduced body weights that persisted from weaning into adulthood (Additional file 1: Figure S1; main factors: diet, *P* < 0.0001, age, *P* < 0.0001; age x diet interaction, *P* < 0.0001).

Significant differences in adiposity index in control mice compared to LUN were detected at 10 and 21 days of age. Females showed 57 % and 50 % (Fig. 2a) reduction of adiposity at 10 and 21 days of age, respectively (main factors: diet, *P* < 0.0001, age, *P* < 0.0001; age x diet interaction, *P* < 0.0001). Similarly, males showed 60 % and 46 % (Fig. 2b) reduction of adiposity at 10 and 21 days of age, respectively (main factors: diet, *P* < 0.001; age x diet interaction, *P* < 0.0001). Uniquely, reduction of adiposity was also observed between 10 and 21 days of age in control males (*P* < 0.01).

### Delayed puberty and changed reproductive performance in F1 progeny undernourished during early postnatal development

The onset of puberty in F1 females, which is typically defined as the time of vaginal opening (VO) and first estrus, was delayed in LUN mice (Fig. 2c). Whereas VO occurred at 27.3 ± 0.4 days of age in CON mice, it occurred at 33.6 ± 0.7 days of age in LUN mice (*P* < 0.0001). Similarly, the mean age of first vaginal estrus (VE) was 34.3 ± 0.6 days in normal fed mice compared with 41.2 ± 0.6 days in LUN mice (*P* < 0.0001). Interestingly, no significant difference in body weights at VO (Additional file 2: Figure S2A) and VE (Additional file 2: Figure S2B) were observed between normally fed animals and LUN mice. Correlation between body weight and age at VO was significant in both normal nourished and underfed mice (*R* = 0.8509, *P* = 0.0004 for CON and *R* = 0.7766, *P* = 0.0018 for LUN; Additional file 2: Figure S2A). Although the correlation between body weight and age at VE was significant in normal fed females (*R* = 0.8458, *P* = 0.0005), it was non-significant in LUN counterparts (*R* = 0.2883, *P* = 0.3395; Additional file 2: Figure S2B).

Not only did changes in neonatal nutrition affect the onset of puberty, they also had long-term effects on later reproductive capability. LUN females displayed a reduced number of estrous cycles, as evaluated by the number of estrus before breeding (4.5 ± 0.2 vs. 3.4 ± 0.2, *P* = 0.0001; Fig. 2c).

The onset of puberty in male F1 progeny, which can be defined as the time of balano preputial separation (BPS), was delayed in LUN males (Fig. 2d). Whereas BPS occurred at 30.2 ± 0.3 days of age in CON mice, it was observed at 35.7 ± 0.6 days of age in LUN mice (*P* < 0.0001). Similar to the results of the experiment in female F1 progeny, no significant difference in body weights at BPS were observed between CON and LUN mice (Additional file 2: Figure S2C). Furthermore, correlation between body weight and age at BPS was non-significant in both CON and LUN males (*R* = 0.2545, *P* = 0.4502 and *R* = 0.4983, *P* = 0.0587, respectively; Additional file 2: Figure S2C).

Percentage of pregnancy was lower in LUN females when compared to CON females bred with CON males (67 % vs. 100 %, 1st breeding; 83 % vs. 100 %, 2nd breeding). Interestingly, CON females bred with LUN males also displayed lower percentage of pregnancy (67 %, 1st breeding; 83 %, 2nd breeding). Although litter sizes at birth did not differ significantly between breeding protocols, substantial improvement in litter size in second pregnancy was observed only in CON females bred with CON males (6.67 ± 0.56 vs. 9.00 ± 0.73; *P* < 0.0457).

### Hypothalamic gene expression networks altered in F1 progeny undernourished during early postnatal development

**Fig. 3:**
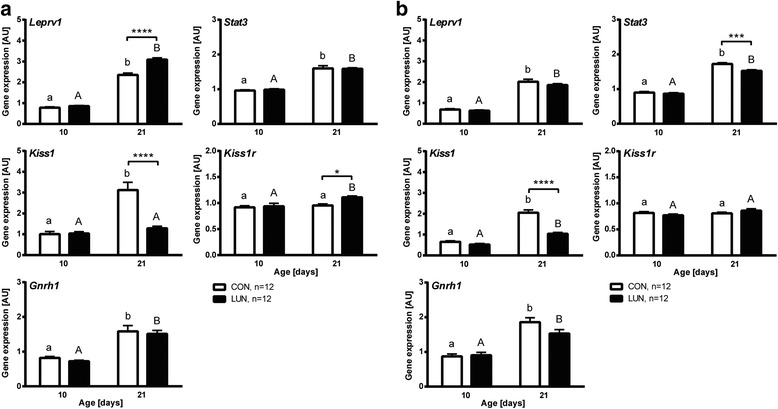
Undernutrition (LUN) from birth to weaning at 21 days of age affects expression of Kisspeptin-signaling related genes in hypothalami of female (**a**) and male (**b**) F1 progeny. Expression levels are presented relative to *Ppib* expression (arbitrary units (AU)) assessed by the real-time RT-PCR. Data are expressed as mean ± SEM. Means with different superscripts differ significantly (small letters – control (CON), capital letters – LUN). Asterisks indicate differences between nutritional protocols for the same day of age (*Two-way ANOVA*; *, *P* < 0.05, ***, *P* < 0.001, ****, *P* < 0.0001)

Because kisspeptins are essential gatekeepers of proper reproductive maturation, including gating by metabolic factors [21] and kisspeptin neural circuits develop gradually during the first weeks of postnatal life [20], we next investigated whether neonatal undernutrition during the neonatal period impacts kisspeptin-signaling related gene expression within the hypothalamus of 10- and 21-day old mice (Fig. 3; Additional file 3: Table S1). While in 10-day old animals there were no diet-dependent differences in leptin receptor (*Leprv1*), signal transducer and activator of transcription 3 (*Stat3*), kisspeptin 1 (*Kiss1*) and its receptor (*Kiss1r*), and gonadotropin releasing hormone 1 (*Gnrh1*) levels, some of these genes were significantly affected by undernutrition 11 days later. Furthermore, though *Kiss1* mRNA expression was down-regulated in both males and females (*P* < 0.0001), *Leprv1* and *Kiss1r* were significantly up-regulated only in females (*P* < 0.0001, *P* < 0.05, respectively). On the other hand down-regulation of *Stat3* expression was only detected in 21-day old males (*P* < 0.001). An age-dependent increase of *Leprv1*, *Stat3*, and *Gnrh1* was observed in both males and females independently of the nutrition protocol (*P* < 0.0001). In contrast, *Kiss1r* mRNA levels were maintained constant only in control animals and LUN males (main factors: diet, *P* < 0.0241, age, *P* < 0.0086; for females only). Uniquely, normal mechanisms for induction of *Kiss1* expression in 21-day old animals was abrogated in both LUN female and male mice (main factors: diet, *P* < 0.0001, age, *P* < 0.0001, age x diet interaction, *P* < 0.0001), with more profound changes observed in females.

**Fig. 4:**
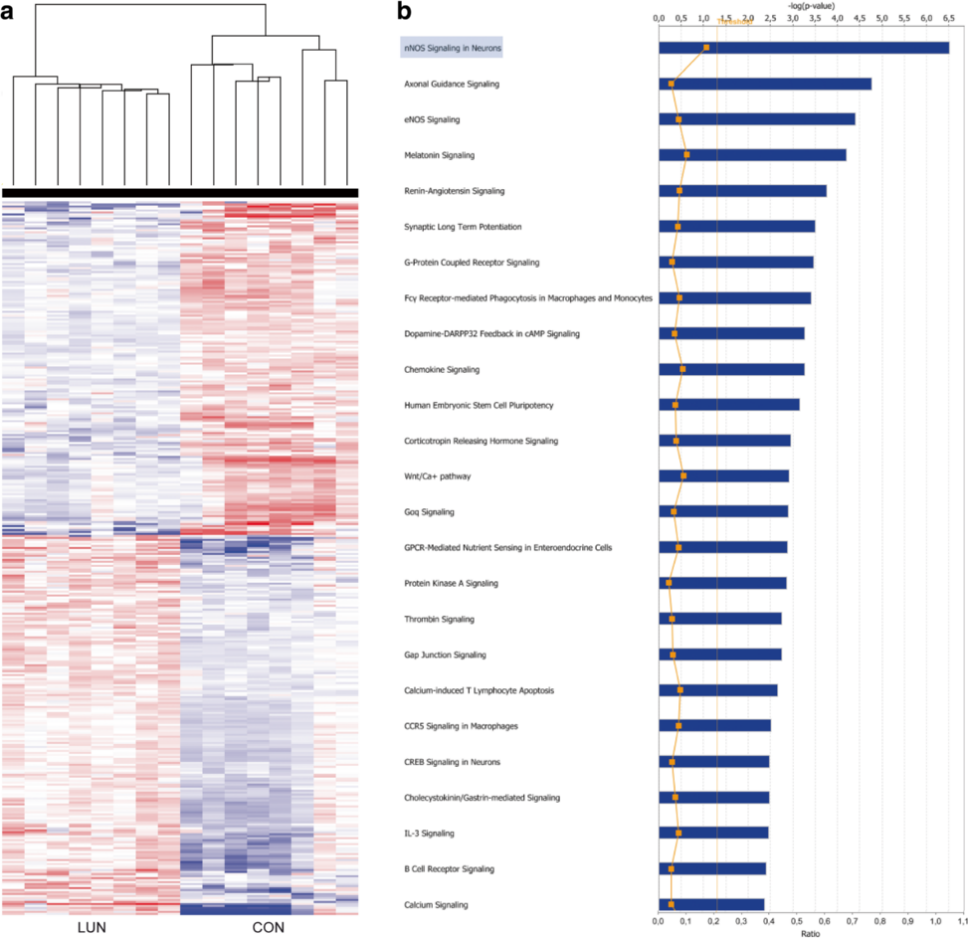
Molecular changes in the hypothalami of 21-day old female F1 progeny undernourished (LUN) from birth to weaning at 21 days of age. **a** Hierarchical clustering of transcriptional profiles, displayed as a heat map of normalized intensity values, from the hypothalamus of control (CON) and LUN mice. Each line represent individual animal. **b** Genes showing differential expression (DEG) classified into 25 Top Canonical Pathways by Ingenuity Pathway Analysis (IPA). The X-axis represents score for the likelihood (−log (Benjamin-Hochberg *p*-value < 0.05)) that genes belonging to a specific functional category are affected in the specific comparisons. The significance cutoff is shown as an orange horizontal line. Ratios (number of affected genes in a given pathway, divided by the total number of genes that make up that pathway) are shown as orange points within the bar. Ratio values are shown on the right y axis

We generated a detailed transcriptional profile of the hypothalamus of 21-days old females, exposed to the control and undernutrition protocols during the lactation period (CON, LUN). The profiles of gene expression in individual samples were determined by microarray analysis. Out of 25,600 well-annotated RefSeq transcripts (over 19,100 unique genes) 16,252 individual sequences were detected. At a false discovery rate of 5 % (−1.5 < fold change > 1.5), 317 genes (165 down- and 152 upregulated) were found to be differentially expressed (Fig. 4a; Additional file 4: Tables S2, Additional file 5: Table S3).

**Fig. 5:**
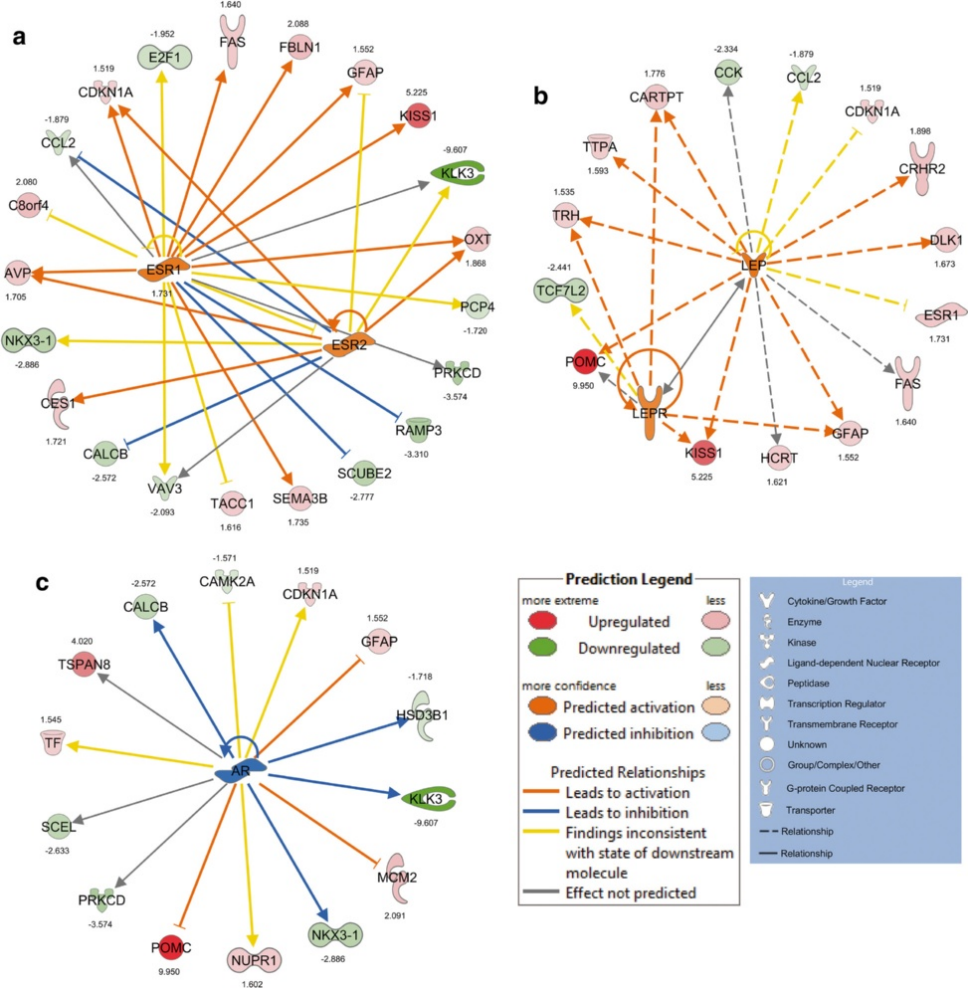
Upstream regulators indicated by IPA potentially affecting expression of DEG in the hypothalamus of 21-day old female progeny undernourished during early development. **a** estrogen receptor 1 and 2 (ESR1 and ESR2), **b** Leptin (LEP), and **c** androgen receptor (AR) were selected for detailed presentation. Networks are displayed graphically as nodes (genes/genes products) and edges (biological relationship between nodes). The node color intensity indicates the fold-change expression of DEG; with red representing up-regulation and green down-regulation in CON vs. LUN animals. The fold change value for individual DEG is indicated under each node. The shapes of nodes indicate the functional class of the gene product and the lines indicate the type of interaction, explained in the legend. All connections were integrated into the computationally generated networks on the basis of the evidence stored in the IPA knowledge memory base indicating relevance for these networks

Using Ingenuity Pathway knowledge base we identified major genetic pathways associated with synthesis of lipids, mass of adipose tissue, quantity of connective tissue, cell cycle progression, and synaptic depression (activation z-score > −/+2.0, Table 1). Among Top 25 Biofunction Categories those responsible for cellular function and maintenance (*p*-values 6.90E-09-6.89E-03), behavior (*p*-values 7.39E-09-6.59E-03), cell-to-cell signaling and interaction and nervous system development and function (*p*-values 7.19E-07-6.89E-03), lipid metabolism, molecular transport, and small molecule biochemistry (*p*-values 1.07E-05-6.02E-03) were identified (Additional file 6: Table S4). Furthermore, genes differentially expressed in the hypothalamus of LUN females were classified into several canonical pathways, including nNOS signaling in neurons (*p*-value 3.11E-07), axonal guidance signaling (*p*-value 1.71E-05), and synaptic long term potentiation (*p*-value 3.19E-04; Fig. 4b-d, Additional file 7: Table S5). In order to explain the observed hypothalamic gene expression changes evoked by undernutrition during early development we performed Upstream Regulator *in silico* search in Ingenuity Knowledge Base using Ingenuity Pathway Analysis (IPA). It allowed us to identify the cascade of potential upstream transcriptional regulators, which could explain the biological activities occurring in the hypothalami of undernourished females (Additional file 8: Table S6). Among top upstream regulators of *Kiss1* expression transcription factor AP-2 alpha (Tfap2a; z-score = 1.966, *P* = 1.15E-03) and estrogen receptor 1 (Esr1, FC = 1.731; z-score = 1.480, *P* = 1.51E-06) were indicated. Because upon hormone activation Esr1 may form a homodimer or a heterodimer with Esr2 (z-score = 1.256, *P* = 1.13E-04) both estrogen receptors were presented as an integrated network (Fig. 5a). Furthermore, both leptin (z-score = 1.604, *P* = 9.52E-04) and its receptor (Lepr; z-score = 1.231, *P* = 2.11E-02) were among upstream regulators of *Kiss1* expression (Fig. 5b). On the other hand, among highly inhibited ligand-dependent nuclear receptors progesterone (PGR; z-score = −1.507, *P* = 1.55E-02) and androgen (AR; z-score = −1.505, *P* = 3.41E-04; Fig. 5c) receptors were found. Interestingly, pro-opiomelanocortin-alpha (*Pomc*; FC = 9.950) and kallikrein 1 (*Klk1*; FC = 9.607), showing opposite profiles of expression, were among genes regulated by AR. Moreover, CCAAT/enhancer binding protein (C/EBP), beta (Cebpb; z-score = 2.331, *P* = 6.94E-04) and GLI family zinc finger 2 (Gli2; z-score = −2.236, *P* = 1.61E-03) were among top upstream transcriptional regulators of molecular programming of hypothalamus.

**Table 1:** Significantly activated IPA BioFunctions identified for DEG in hypothalamus of CON vs LUN 21-day old female progeny (F1)

Categories	Functions annotation	*p*-value	Predicted activation state	Activation z-score^a^	Bias-corrected z-score	Molecules*
Lipid metabolism, small molecule biochemistry	synthesis of lipid	0.00414	Increased	2.064	2.121	**ACSL3**, **AGTR1**, **AVP**, CCL2, **CES1**, **CRHR2**, **DPP4**, **ESR1**, **FAS**, FCGR1A, HSD3B1, **KISS1**, **MARC2**, MGST2, **NR1H3**, **OXT**, **PIK3R1**, **POMC**, PRKCD, PROX1, PTK2B, RGS3, TCF7L2, **TRH**
Connective tissue development and function, tissue morphology	mass of adipose tissue	0.00097	Decreased	−2.871	−2.857	**CARTPT**, **CDKN1A**, **DLK1**, E2F1, **ESR1**, FOXP2, HRH3, **NR1H3**, **POMC**, SHOX2
	quantity of connective tissue	0.00083	Decreased	−2.179	−2.161	ADAMTS1, **Ahsp**, CALCB, **CARTPT**, CCL2, **CDKN1A**, **CES1**, CHRNA7, **CIDEB**, **CRHR2**, E2F1, **ESR1**, **HBA1/HBA2**, **Hbb-b1**, HRH3, KLK3, **NR1H3**, **PIK3R1**, **POMC**, **PRKG1**, **SLC14A1**, **SLC4A1**, **UHRF1**
Cell cycle	cell cycle progression	0.00057	Decreased	−2.319	−2.289	ADARB1, **AVP**, CALCB, CAMK2A, CCK, **CDKN1A**, **DPP4**, E2F1, **ESR1**, **FAS**, **HSPA1A/HSPA1B**, **ING3**, LEF1, **MCM2**, MYOCD, NKX3-1, **NUPR1**, **NUSAP1**, PGAM2, **PIK3R1**, PRKCD, PRKCG, PRKCH, **PRKG1**, PROX1, PTK2B, PTPRN2, RASSF3, RBM3, TCF7L2, **TF**, **USP1**, USP2, VAV3, ZIC1
Cell-to-cell signaling and interaction	synaptic depression	0.00017	Decreased	−2.200	−2.171	ADCY1, CAMK2A, **CPEB3**, DLG4, GRID2IP, MAP1B, PRKCG, PTK2B, SV2B

^a^Threshold for activation z- score for decreased BioFunctions was maintained at ≤ −2.179

*Genes up- (bold) and down-regulated in hypothalami of control (CON) vs. LUN 21-day old F1 females

**Fig. 6:**
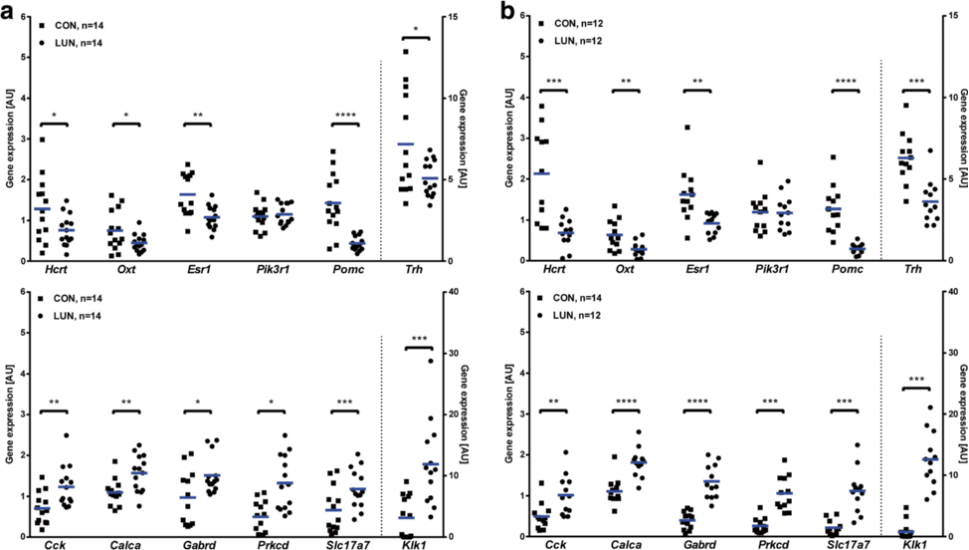
Relative expression of genes participating in molecular changes occurring in the hypothalami of 21-day old female (**a**) and male (**b**) F1 progeny. Gene expression analyses were performed using qRT-PCR. Expression levels are presented relative to *Ppib* expression (arbitrary units (AU)). Asterisks indicate difference between means (blue line; *t test*; *, *P* < 0.05, **, *P* < 0.01, ***, *P* < 0.001, ****, *P* < 0.0001). CON – F1 control progeny, LUN – F1 progeny undernourished during lactation

Validation of microarray results using qRT-PCR method confirmed that the expression of hypocretin (*Hcrt*), oxytocin (*Oxt*), thyrotropin releasing hormone(*Trh*), *Pomc*, and *Esr1* was decreased in the hypothalamus from 21-day old LUN females (Fig. 6a, upper panel). On the other hand, expression levels of cholecystokinin (*Cck*), calcitonin/calcitonin-related polypeptide, alpha (*Calca*), gamma-aminobutyric acid (GABA) A receptor, subunit delta (*Gabrd*), protein kinase C, delta (*Prkcd*), solute carrier family 17 (sodium-dependent inorganic phosphate cotransporter), member 7 (*Slc17a7*), and *Klk1* were maintained at higher levels in LUN females (Fig. 6a, lower panel). Expression profile of phosphatidylinositol 3-kinase, regulatory subunit, polypeptide 1 (*Pik3r1*) did not overlap between these two methods. In addition, expression of tested genes in the hypothalamus of males exposed to undernutrition during lactation was validated by qRT-PCR (Fig. 6b) and showed similar profiles as for females.

### Maternal diet during lactation affects body composition of 2^nd^ generation offspring

**Fig. 7:**
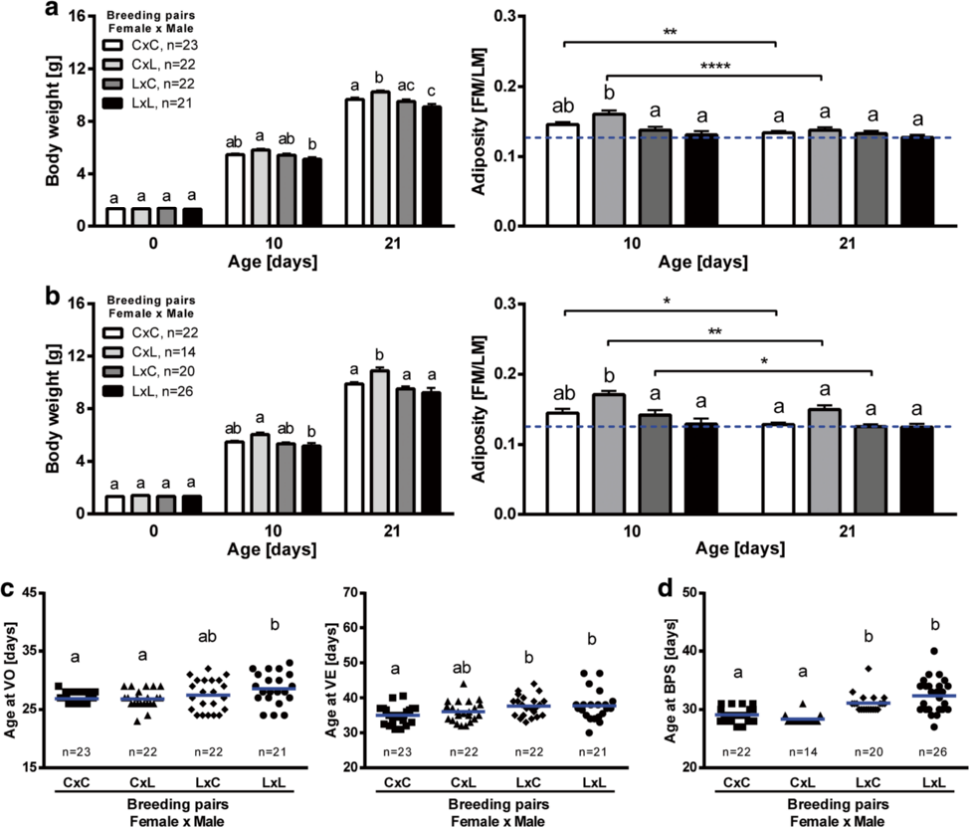
Undernutrition (LUN) in F1 generation affects body composition and puberty attainment in F2 offspring. Body weight and adiposity index (fat mass/lean mass [FM/LM]) in female (**a**) and male (**b**) progeny. Data are expressed as mean ± SEM. Means with different superscripts differ significantly within the same day of age. Asterisks indicate difference between days in each breeding protocol (*Two-way ANOVA*; *, *P* < 0.05, **, *P* < 0.01, ****, *P* < 0.0001). All comparisons of body weights between days in each breeding protocol (CxC, CxL, LxC, LxL) were significant (*P* < 0.0001). Timing of vaginal opening (VO; **c**), vaginal estrus (VE; **c**) and balano preputial separation (BPS; **d**) in F2 progeny, generated in different breeding protocol. Means (blue line) with different superscripts differ significantly (statistical significance was calculated by *One-way ANOVA*). C – CON (F1 control progeny), L – LUN (F1 progeny undernourished during lactation)

To investigate the multigenerational impact of malnutrition during early development in the next experiment we evaluated changes in body composition, puberty attainment and patterns of gene expression in the hypothalami of F2 progeny generated from F1 control (CON = C) and undernourished (LUN = L) mice. In our mouse model, F1 mice were bred according to their nutritional protocol to generate F2 progeny originating from control (C♀ x C♂), mixed (C♀ x L♂, L♀ x C♂), and undernourished (L♀ x L♂) parents. At first breeding, F1 LUN females (day 55) and males (day 67) showed lighter body weight compared with CON mice (Additional file 1: Figure S1) and this change in body weights persisted in pregnant females, as measured on day 10 of pregnancy (1st gestation: 20.66 ± 0.24 vs. 21.78 ± 0.29, *P* < 0.008; 2nd gestation: 27.54 ± 0.32 vs. 29.33 ± 0.59, *P* < 0.016). Consequently, we observed that undernutrition of either one or both parents during lactation was associated with changed growth rates of female and male pups. As early as day 10, females (Fig. 7a, *P* < 0.001) and males (Fig. 7b, *P* < 0.05) generated from both undernourished parents (LxL) exhibited a lighter body weight compared with CxL mice and this change in body weight persisted at weaning (*P* < 0.0001; main factors: breeding protocol, *P* = 0.0002 for females and *P* = 0.002 for males, age, *P* < 0.0001; age x breeding protocol interaction, *P* < 0.0001). Interestingly, at weaning, body weight of CxL mice was significantly different also from CxC and LxC animals (*P* < 0.001 and *P* < 0.05, respectively). Differences between body weight of F2 progeny generated from control (CxC) and undernourished (LxL) parents was only indicated in females at weaning (*P* < 0.05).

Significant differences in adiposity index between F2 mice generated by different dietary protocols were only seen at 10 days of age (Fig. 7a, b). Both CxL females and CxL males showed increased adiposity when compared to LxC and LxL mice (*P* < 0.05 and *P* < 0.0001, respectively; main factors: breeding protocol, *P* = 0.004 for females and *P* = 0.0007 for males, age, *P* < 0.0001; age x breeding protocol interaction, *P* = 0.0016 for females). Reduction of adiposity between 10 and 21 days of age was observed only for CxC (*P* < 0.01) and CxL (*P* < 0.0001) females (Fig. 7a). All males, except for LxL, showed significant reduction of adiposity between 10 and 21 days of age (*P* < 0.05; Fig. 7b).

### Maternal diet during lactation delays puberty attainment of 2^nd^ generation offspring

Similar to the initial experiments on F1 progeny, F2 progeny was tested for onset puberty using the same parameters. The most striking observation was that both female and male F2 progeny showed delayed puberty when only mother (LxC) or both parents (LxL) were undernourished during lactation (Fig. 7c, d). Whereas VO occurred at 26.9 ± 0.2, 26.8 ± 0.3 and, 27.5 ± 0.3 days of age in CxC, CxL and LxC mice, respectively, it was observed at 28.6 ± 0.6 days of age in LxL females (Fig. 7c; *P* < 0.05). The mean age of VE was 35.0 ± 0.5 and 36.1 ± 0.6 days in CxC and CxL mice, respectively, compared with 37.7 ± 0.6 and 37.7 ± 0.9 days in LxC and LxL mice, respectively (*P* < 0.05). Interestingly, no significant difference in body weights at VO (Additional file 9: Figure S3A) were observed, but it was significantly increased in LxC females at VE (vs. CxC, *P* < 0.01; Additional file 9: Figure S3B). Correlations between body weight and age at VO was significant in CxL, LxC and CxC females (*R* = 0.7968, *P* < 0.0001, *R* = 0.7613, *P* < 0.0001 and *R* = 0.7996, *P* < 0.0001, respectively; Additional file 9: Figure S3A). Although the correlation between body weight and age at VE was significant in CxC females (*R* = 0.7911, *P* < 0.0001), it was non-significant in CxL, LxC and CxC females (*R* = 0.322, *P* = 0.1437, *R* = 0.3897, *P* = 0.073 and *R* = 0.2999, *P* = 0.1865, respectively; Additional file 9: Figure S3B).

The onset of puberty in male F2 progeny was earlier in CxC and CxL males (Fig. 7d). Whereas BPS occurred at 29.1 ± 0.3 and 28.4 ± 0.2 days of age in CxC and CxL mice, respectively, it was observed later at 31.1 ± 0.4 and 32.4 ± 0.6 days of age in LxC and LxL males (*P* < 0.001). Interestingly, significant increase in body weight at BPS was observed in LxL males (vs. CxC, *P* < 0.001; Additional file 10: Figure S4). Furthermore, a weak or no correlation between body weight and age at BPS was detected in male F2 progeny generated in each breeding protocol (CxC, CxL, LxC, LxL; *R* = 0.6949, *P* = 0.0003, *R* = −0.1928, *P* = 0.5089, *R* = 0.4566, *P* = 0.0430, *R* = −0.3464, *P* = 0.0830, respectively; Additional file 10: Figure S4).

### Maternal diet during lactation change hypothalamic gene expression networks of 2^nd^ generations

**Fig. 8:**
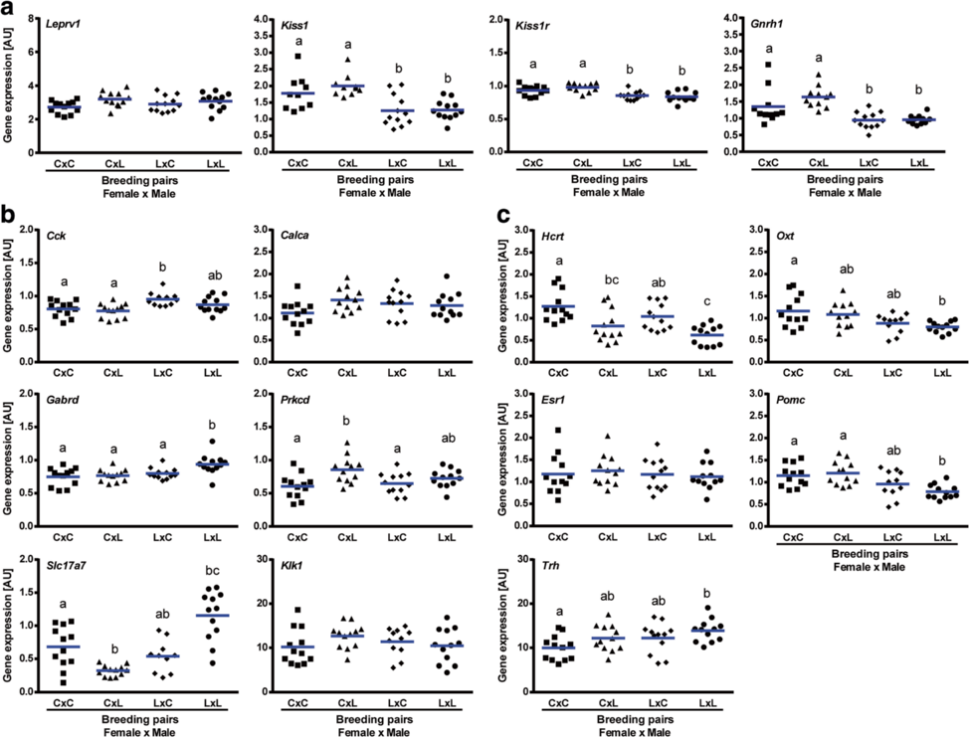
Molecular changes in the hypothalami of 21-day old female F2 progeny. **a** Expression pattern of Kisspeptin-signaling related genes. Genes showing increased **b** and decreased **c** expression in hypothalami of F1 undernourished neonates were tested in F2 females. Expression levels are presented relative to *Ppib* expression (arbitrary units (AU)). Means (blue line) with different superscripts differ significantly (statistical significance was calculated by *One-way ANOVA*; *n* = 12/breeding protocol (CxC, CxL, LxC, LxL)). C – CON (F1 control progeny), L – LUN (F1 progeny undernourished during lactation)

**Fig. 9:**
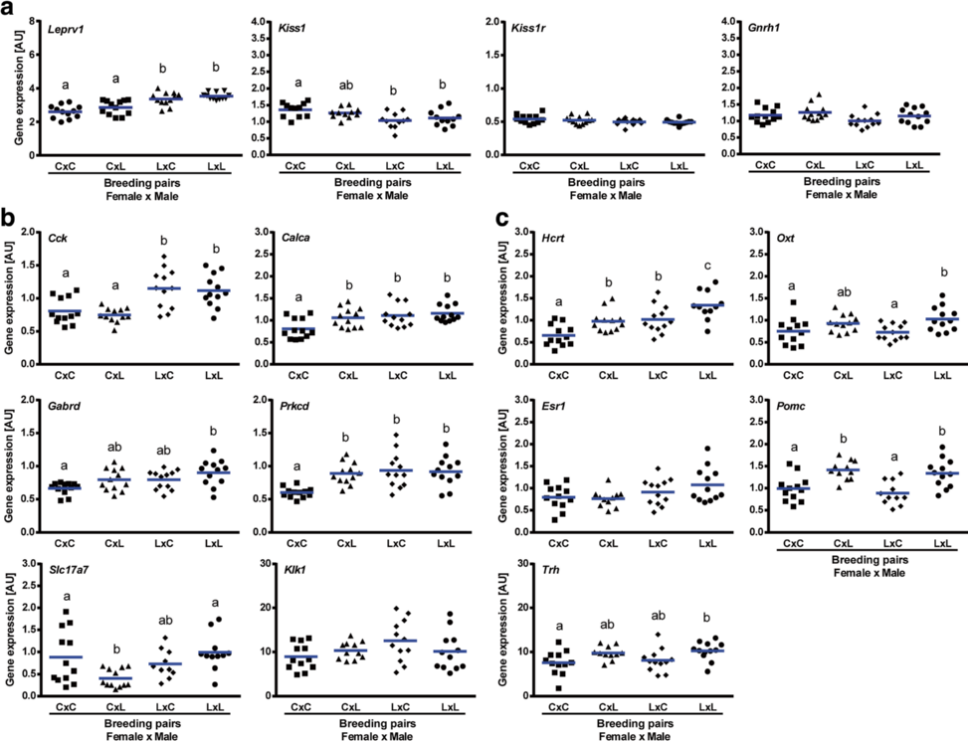
Molecular changes in the hypothalami of 21-day old male F2 progeny. **a** Expression pattern of Kisspeptin-signaling related genes. Genes showing increased **b** and decreased **c** expression in the hypothalami of F1 undernourished neonates were tested in F2 males. Expression levels are presented relative to *Ppib* expression (arbitrary units (AU)). Means with different superscripts differ significantly (statistical significance was calculated by *One-way ANOVA*; *n* = 12/breeding protocol (CxC, CxL, LxC, LxL)). C – CON (F1 control progeny), L – LUN (F1 progeny undernourished during lactation)

Since mice undernurtured before weaning by reduced food availability give birth to progeny showing signs of delayed puberty, we hypothesized that the expression of genes in the hypothalamus of progeny from undernourished parents will also be affected. To test this hypothesis we performed qRT-PCR on the hypothalamus of 21-day old F2 progeny. Kisspeptin-signaling related genes expression within the hypothalamus of 21-day old mice is presented in Figs. 8a and 9a and Additional file 3: Table S1. One-way ANOVA showed sex specific patterns of expression of *Leprv1*, *Kiss1r*, and *Gnrh*. Although *Kiss1* mRNA levels were downregulated in LxC and LxL F2 progeny irrespective of sex (*P* < 0.05), *Leprv1* expression was upregulated only in LxC and LxL F2 males (vs. CxC, *P* < 0.001). On the other hand, *Kiss1r* and *Gnrh* mRNA was decreased only in LxC and LxL F2 females (vs. CxC, *P* < 0.05). Noteworthy, kisspeptin-signaling related genes showed altered gene expression in sexually mature F2 females (Additional file 11: Figure S5). Levels of *Leprv1*, *Kiss1*, and *Gnrh* mRNA were maintained high in hypothalami collected from LxL F2 females at diestrus (vs. CxC, *P* < 0.01, *P* < 0.001, *P* < 0.05, respectively).

Amongst genes upregulated in the hypothalamus of F1 undernourished progeny only *Cck* and *Gabrd* showed increased expression either in both or only one out of LxC and LxL F2 progeny (Figs. 8b and 9b). Levels of *Cck* were significantly higher in both LxC and LxL males (vs. CxC, *P* < 0.01) and only LxC females (vs. CxC, *P* < 0.05). Upregulated expression of *Gabrd* was observed in both LxL females and males (vs. CxC, *P* < 0.01). Interestingly, the lowest expression of *Slc17a7* was observed in CxL mice regardless of sex (vs. CxC, *P* < 0.05). Despite a similar pattern of *Calca* and *Prkcd* expression in hypothalami of CxL, LxC and LxL males (vs. CxC, *P* < 0.05 and *P* < 0.01, respectively) it was maintained constant (*Calca*) or slightly regulated in F2 females (*Prkcd*; CxL vs. CxC and LxC, *P* < 0.01 and *P* < 0.05, respectively). Our data showed no differences in *Klk1* levels between breeding protocols irrespective of breeding protocol.

In addition, genes showing decreased expression in hypothalami of undernourished F1 progeny were tested in F2 animals (Figs. 8c and 9c). Noteworthy, sex specific patterns of *Hcrt* expression was observed in F2 progeny, with decreased levels in CxL and LxL females (vs. CxC, *P* < 0.01 and *P* < 0.0001, respectively) and increased in CxL, LxC, and LxL males (vs. CxC, *P* < 0.05, *P* < 0.01 and *P* < 0.0001, respectively). While *Oxt* and *Pomc* mRNA expression was maintained low in LxL females (vs. CxC, *P* < 0.01) it was upregulated in LxL males (vs. CxC, *P* < 0.05). Similarly, increased expression of *Trh* was maintained in LxL F2 progeny regardless of sex (vs. CxC, *P* < 0.05). *Esr1* expression was maintained constant irrespective of breeding protocol.

## Discussion

Undernutrition of lactating mothers perturbs morphological and molecular postnatal development of their progeny. Here, we show that this nutritional perturbation of development extends to the expression of genes responsible for hypothalamic neural circuits controlling reproductive function in offspring of the first (F1) and the following (F2) generation. Dysregulation of kisspeptin- related gene expression in the hypothalamus can be, in part, responsible for delayed puberty attainment in F1 and F2 male and female progeny. Moreover, the analysis of the hypothalamic transcriptome has led to the identification of genetic pathways and functions associated with behavior (e.g. grooming, maternal behavior, and sexual receptivity of female organism), neural networking (e.g. nNOS signaling in neuros, axonal guidance signaling, synaptic long term potentiation, neurotransmission) and endocrine function (e.g. glucose tolerance, insulin sensitivity, kisspeptin signaling, and steroid hormone production). Upstream regulators analysis revealed that leptin and ligand-depended nuclear receptors, such as androgen and estrogen receptors, can be involved in programming the onset of puberty. Hence, our data strongly suggest that undernutrition during critical stages of development leads to a sex-specific molecular programming of the hypothalamus that is subsequently transmitted to male and female progeny in subsequent generations.

Our morphometric data, registered up to approximately 6–7 weeks after the experimental period of undernutrition during suckling, indicate that the undernourished female and male progeny have not recovered and that their weight is significantly less than those of the controls. Adiposity, measured at 10 and 21 days of age was also much lower in LUN than in CON mice, is in agreement with our previous studies [19]. Engelbregt and co-workers [22] showed that postnatal food restriction, achieved by litter enlargement, led to a significantly lower percentage of fat in female and male progeny at puberty. Maternal undernutrition during pregnancy also affects body composition, as lean and fat masses were significantly reduced in F1 mice exposed to low protein diet *in utero* [23] and absolute amounts of fat were decreased in intrauterine growth restricted females [22]. Interestingly, in our present study body weights and composition of second generation mice, never exposed directly to postnatal undernutrition, were also significantly reduced. This data indicates that presence of a negative energy balance not only affected body composition of the F1 generation, but also that of the phenotype of F2 male and female progeny in sexually dimorphic manner. Although, adiposity was only increased in 10-day old CxL male and female offspring, at weaning body weights were increased for CxL male and female offspring and decreased for LxL females.

Other investigators have previously shown that nutritional stress during early stages of development (mainly intrauterine), including low-protein or energy diet, and high-fat feeding, can affect the onset of puberty and reproductive capacity [18, 24–28]. Frisch and Revelle [29, 30] already in the early 1970 proposed the critical *weight/fat mass hypothesis* suggesting that a weight of 48 kg or 22 % body fat allows puberty to start. Indeed, the timing of the onset of puberty appears to be closely associated with body weight and adiposity in many species. For instance, sufficient levels of fat are essential for initiating puberty in rodents and humans [31, 32]. On the other hand, obesity was shown to perturb various aspects of pubertal development [14] Although both F1 males and females had comparable body weights at puberty, irrespective of nutritional protocol, only females showed a strong correlation between the timing of sexual maturation, i.e. the age at vaginal estrus and body weight. This was not observed in LUN counterparts, which have altered body composition and changed hypothalamic gene expression controlling neural circuits. Although we did not measure adiposity at puberty, it needs to be stressed that our previous studies showed that LUN mice not only maintain lower body weights till adulthood but also reduced fat and lean mass, as well as leptin levels [19]. Interestingly, similar loss of correlation between age at vaginal estrus and body weight was indicted for CxL, LxC and LxL F2 females. Moreover, parental nutritional history seems more likely to be involved in puberty onset programming in F2 generation, since progeny of either both LUN parents or only the LUN mother showed delayed puberty. Thus, our results support a model in which malnutrition experienced during early postnatal development programs the recapitulation of the phenotype in the second generation, having consequences on the age of onset and rate of progression through puberty.

Although the timing of puberty is influenced by several factors including nutrition and adiposity [33] as well as size at birth [34], it also has a strong and obligatory endocrine component. Puberty is triggered by reactivation of the HPG axis which stimulates the release of LH and FSH from the pituitary gland, which in turn induces gonadal maturation [35]. The hypothalamic kisspeptin system has been suggested to play a major role in pubertal activation [36] and kisspeptin neural circuits develop gradually during the first weeks of postnatal life in mice [20]. We found that the induction of genes involved in kisspeptin-signaling, i.e. *Leprv1*, *Stat3*, and *Gnrh1* occurs between 10 and 21 days of age and sex specific patterns are characteristic for *Leprv1* and *Stat3*. Moreover, expression of *Kiss1* in F1 progeny of both sexes showed significant reduction in response to undernourishment during the lactation period, while *Kiss1r* was slightly increased only in 21-day old F1 females. A similar response to fasting was showed in adult animals, in which decreased hypothalamic KISS1 expression was accompanied by lower circulating levels of LH [37]. Nevertheless, increased expression of *Kiss1r* may suggest a kind of compensation mechanism occurring at this time point only in females, leading to upregulation of *Kissr* expression. Interestingly, downregulation of Kiss1 but upregulation of Kiss1r mRNA was observed in fasted prepubertal male and female rats [37]. The hypothalamus is structurally and functionally sexually dimorphic [38]. Sexual differentiation of the hypothalamus is strongly influenced by differential exposure of male and female brains to sex steroid hormones in early life [39]. Interestingly, GnRH neurons do not express most sex steroid hormone receptors [40], thus neural circuits are developed *via* afferent inputs to GnRH neurons and include the action of kisspeptin. Expression of Kiss1 in the arcuate nucleus (ARC) showed apparent sex differences both at birth and during early postnatal life in rodents [41, 42]. This perinatal sex difference in ARC Kiss1 parallels the sex difference in serum gonadotropins at this time, with females having higher FSH and LH than males [42, 43]. Importantly, the effect of neonatal undernutrition on kisspeptin-signaling related genes within the hypothalami, evident in both sexes, persisted over the generations, with similarly reduced *Kiss1* expression in LxC and LxL F2 progeny of both sexes. Sex differences, however, were seen for *Leprv1* (increased in LxC and LxL F2 males)*, Kiss1r* and *Gnrh1* (decreased in LxC and LxL F2 females). It is interesting to note that F2 generation females showed transcriptomic changes at reproductive maturity, but only those generated from both undernourished parents (LxL) showed increased expression of *Leprv1*, *Kiss1*, and *Gnrh1* at diestrus.

The comparative microarray analysis of hypothalamus of 21-day old F1 females revealed more profound transcriptomic changes influencing development of hypothalamus evoked by lactation undernutrition during suckling. We found that several genes involved in nervous system development and function, e.g. *Hcrt* [44], *Oxt* [45], *Esr1* [46], *Pomc* [47], *Trh* [48], *Cck* [49], *Calca* [50], *Gabrd* [51], and *Slc17a7* [52] have similar patterns of expression in LUN F1 males and females. Among them *Oxt*, *Esr1*, and *Gabrd* have known roles in reproductive system development and function, proved by our Ingenuity Knowledge Base search. As mentioned above, careful attention needs to be paid to the steroids and their role in development of neuronal circuity, i.e. negative and positive feedbacks, leading to puberty attainment and influencing subsequent reproductive performance. Although in anteroventral periventricular nucleus and neighboring rostral periventricular nucleus (AVPV/PeN) KISS expressing cells also moderately co-express ESR2, at least in females, ESR1 is believed to be the primary estrogen receptor for upregulating *Kiss1* expression, since estradiol (E2) treatment does not stimulate AVPV/PeN *Kiss1* expression in ESR1 KO mice, but still does it in ESR2 KO mice [53]. In contrast, kisspeptin neurons in the ARC are negatively regulated by E2 in both male and female rodents [53, 54]. Moreover, testosterone can also regulate *Kiss1* expression in the ARC [54] *via* AR, which was detected in over 60 % of Kiss1 expressing cells in the ARC of male mice [53, 54]. Both ligand-depended nuclear receptors, ESRs and AR, were also identified among the upstream transcriptional regulators of molecular programming of hypothalamus in our study. Recently it was suggested that pre-weaning levels of circulating E2 are required to keep the reproductive axis at bay until central inhibitory mechanisms are fully developed, or until the development of hypothalamic circuitry capable of reacting to E2 with both negative and positive feedback responses [55]. Moreover, AVPV/PeN and ARC kisspeptin neurons receive GABAergic synaptic input [56] and GABAergic transmission to ARC kisspeptin neurons is modulated by E2 in female mice [57]. Our observations are thus the first to suggest possible interactions of Esr1, kisspeptin-signaling genes (namely *Kiss1* and *Kiss1r*), and GABAergic transmission to hypothalamic kisspeptin neurons in hypothalamic gene networks, impaired by malnutrition during critical stages of development of F1 offspring. In contrast to F1 males and females, F2 offspring showed sex and parental nutritional history dependent profiles of mRNA expression at 21 days of age. Regardless of sex, similar patterns of expression were seen for *Thr*, *Gabrd*, and *Esr1*, however, *Esr1* was maintained constant in all tested breeding protocols, suggesting different mechanisms associated with programming of hypothalamic neural circuity in F2 offspring.

Among factors regulating onset of puberty leptin is thought to be a permissive neuro-regulatory factor determining onset of puberty in several mammalian species [58]. It synchronizes growth, reproductive maturity and fertility with periods of food availability [59]; post-weaning treatment with leptin accelerates puberty in rodents [60], and leptin in the rat neonate affects hypothalamic maturation [61]. Nevertheless, recent studies have demonstrated that leptin transfers information about metabolic status to kisspeptin neurons indirectly because leptin administration fails to phosphorylate STAT3, a key component of a major intracellular signaling pathway mediating leptin action [62]. Indeed, leptin receptors are located on many neuronal populations in the hypothalamus [63] which can cross-talk with kisspeptin neurons [64, 65]. The power of the mouse model for studies of nutritional programming is that development of white adipose tissue occurs exclusively during the postnatal period, and levels of plasma leptin are almost completely determined by leptin secreted by the inguinal fat [19]. While it is well known that maternal undernutrition or protein deficiency leads to underweight pups at birth and a shift in the leptin surge, we previously showed severely suppressed levels of plasma leptin in pups and adults exposed to undernutrition during the postnatal period [19]. Since in the present study neonatal growth trajectories, including body composition, in F1 progeny under-nourished during lactation began to deviate from those of control pups at the time that coincides with neonatal surge in circulating leptin, this hormone may be a central driver in initiating hypothalamic neural circuity development in newborn pups. Previous results showed that postnatal food restriction, achieved by litter enlargement, leads to altered leptin regulation and hypoleptinemia at puberty [15, 22]. Since attenuation in the development of the neuronal feeding circuitry in the hypothalamus has been proposed to be the basis for the hyperphagic and reproductive phenotypes of leptin deficient *ob/ob* mice [61], the severe suppression of circulating leptin (>90 %) in LUN mice at 10 days of age is expected to have profound impacts on the development of regulatory neuronal circuits regulated by leptin, including the reproductive axis. Leptin involvement in hypothalamic gene networks was also proved in our *in silico* analysis where it was identified as the powerful upstream transcriptional regulators of molecular programming of hypothalamus in F1 female progeny, leading to altered expression of several genes, including *Pomc*, *Kiss1*, *Cck*, CART prepropeptide (*Cartpt*), *Hcrt*, *Trh*, and *Esr1*. It was striking that some of these genes were also affected in F2 female and male progeny never exposed directly to undernutrition and the accompanying leptin deficiency. Similar downregulation of *Kiss1* hypothalamic expression was detected in F2 offspring generated from either lactation undernourished F1 female and control F1 male (LxC) or both undernourished F1 parents (LxL). Other molecular changes occurring in the F2 hypothalamus appeared to be sexually dimorphic and dependent on paternal nutritional history, as opposite expression of *Oxt*, *Pomc* and *Hcrt* was observed in LxL F2 females and males, but only when compared to CxC F2 counterparts.

Besides Kiss1 neurons, other hypothalamic populations may also provide afferent signals on the HPG axis to regulate reproduction. Importantly, ARC POMC neurons comprise a critical metabolic-sensing pathway controlling the reproductive neuroendocrine axis [66]. POMC neurons are well positioned to be a direct target of leptin and insulin, as they express LEPRs [67] and insulin receptors (IRs) [68]. Consequently, POMC neurons as a target of leptin/insulin actions were shown to be important for fertility, as IR/LEPR-POMC KO mice exhibit lengthened reproductive cycles, follicular arrest, hyper-androgenemia, and infertility [69]. Importantly, *Kiss1* expressing cells were shown to communicate with neuropeptide-Y (NPY) and POMC cells, suggesting that these three cell types coordinate brain control of reproduction and metabolic homeostasis [64, 65]. In addition, leptin was shown to inhibit HCRT neurons both directly and indirectly by several GABA-dependent as well as -independent mechanisms [70, 71]. Thus, characteristic hypothalamic expression patterns of *Kiss1*, *Gabrd, Pomc,* and *Hcrt* in F1 undernourished offspring, determined by parental nutritional history and sex specificity in the following F2 generation clearly support the concept that reciprocal connections exist between these factors that are responsible for the developmental programming of hypothalamic circuitry controlling reproductive function in a multigenerational manner.

## Conclusions

In conclusion, the first weeks of postnatal life when neurons send their axonal projections to their target sites are recognized as the second important developmental period for hypothalamic development in rodents [72]. We showed that this early developmental plasticity of hypothalamus leads to transcriptomic changes in hypothalamus when challenged with undernutrition and in consequence change body composition and timing of puberty in F1 generation. This work also demonstrates that malnutrition during critical stages of development leads to sex-specific molecular programming of hypothalamus that is subsequently transmitted to both male and female F2 progeny, defining their phenotype dependent on parental nutrition history. It seems likely that epigenetic mechanism is involved in molecular programming of HPG axis, since histone deacetylases (HDACs) were among potent upstream regulators of the molecular changes occurring in F1 females. Other studies showing: 1) protective effect of leptin against the later obesity *via* changes in promoter methylation of some genes, including the hypothalamic *Pomc* [73] and 2) histone deacetylation and DNA methylation of Kiss1 in AVPV/PeN contributing to sex-specific differences in the neuronal cell number [74] give a rationale for further testing of our hypothesis. These observations open up new roads for the investigation of mechanisms involved in multigenerational molecular programming of the in hypothalamic neural circuits controlling reproductive function in both sexes, including deep investigation of molecular changes in specific hypothalamic nuclei (ARC, AVPV/PeN) having important roles in the control of reproductive function and containing distinct populations of kisspeptin neurons.

## Methods

### Animal care and experimental design

C57BL/6 J breeders were obtained from the Jackson Laboratory (Bar Harbor, Maine, United States) and maintained at Pennington Biomedical Research Center as described (19). Newborn F1 mice were raised from birth to weaning with one of two sets of nutritional conditions (Fig. 1, upper panel): 1) the control condition (CON) had 8 pups per litter and the mother was fed the Breeders Chow diet 5015 (22 kcal % fat) *ad libitum*; 2) for lactation undernutrition condition (LUN) litter size was fixed at 8 pups, but the mother was only fed 50 % of the food (LabDiet 5015) consumed by the control mice the previous 24 hr [19]. After weaning the F1 offspring from the all nutritional conditions were treated the same; from weaning until approx. 8 wks of age mice were fed Purina LabDiet 5053 (11 Kcal % fat) *ad libitum*. From weaning until 8–9 wks of age male and female progeny were housed separately (3–5 mice per pen) for the remainder of the experiment. To generate the second-generation offspring (F2), unrelated nonsibling F1 CON (C) and LUN (L) females (♀) and males (♂) were mated at age 2 months in four combinations: C♀ x C♂, C♀ x L♂, L♀ x C♂, and L♀ x L♂ (Fig. 1, lower panel). Lactating F1 females were not subjected to food restriction. At birth, F2 litters were adjusted to eight pups per dam, and all mice had free access Breeders Chow diet 5015 until weaning. Similarly, from weaning F2 male and females were housed separately (3–5 mice per pen) for the remainder of the experiment and fed Purina LabDiet 5053 *ad libitum*. In F1 generation data were collected from 4 CON and 8 LUN litters. In F2 generation In F2 generation data were collected from at least two litters generated by each breeding pair (CxC, *n* = 6; CxL *n* = 6; LxC, *n* = 6; LxL, *n* = 7). All protocols have been approved by the Pennington Biomedical Research Center’s Institutional Animal Care and Use Committee.

### Tissue processing

Mice were anesthetized with isoflurane until the toe-pinch withdraw reflex and corneal reflex was lost. After cervical dislocation, the brain was removed and placed on an ice-cooled glass plate with the cortex facing down. The hypothalamus was dissected along the following boundaries: laterally 2 mm either side of the third ventricle, 2 mm dorsally from the base of the brain and rostrocaudally from the optic chiasm to the posterior border of the mammillary bodies. Dissected hypothalami were stored at −80 °C until further use.

### Phenotypic assays

#### Body composition

Adiposity was determined from body weight and body composition measurements performed by nuclear magnetic resonance (NMR, Bruker, The Woodlands, Texas, United States) at days 10 and 21 of postnatal development in F1/F2 female and male progeny. Body weights were recorded at birth (day 0), on day 10 and 21 postpartum, and was continued until about day 55 (for F1 females) or 67 (for F1 males) of age.

#### Puberty attainment

The effects of changes in postnatal feeding on the timing of puberty were evaluated in F1/F2 female and male progeny. Age of vaginal opening (VO), vaginal estrous (VE) or balano preputial separation (BPS), as consensus external markers of puberty in female and male rodents, respectively, were monitored daily (08:00 hr) starting from day 23 postpartum. Onset of puberty was defined as the age (in days) in which VO or BPS occurred. Cyclic stages of the ovaries were studied by daily vaginal smears after vaginal opening until about day 55 of age. Body weight at VO, VE and BPS for each nutritional protocol (F1 CON and LUN progeny) and each breeding protocol (F2 CxC, CxL, LxC, and LxL progeny) was assessed to evaluate association between body weight and age at VO, VE and BPS in F1/F2 females and males, respectively.

### Transcriptomic analysis

#### Real-time RT-PCR

Total RNA was isolated from hypothalamus using TRI Reagent and BCP phase separation reagent (Molecular Research Center Inc. Cincinnati, OH, USA). RNA was further purified by using the RNAeasy minikit (QIAGEN, Valencia, CA, USA) and stored at −80 °C in RNase-free water with addition of SUPERase-In (Ambion, Austin, TX, USA) for RNA protection. Quality and quantity of RNA was determined using UV spectrophotometry (Nanodrop) and Agilent 2100 Bioanalyzer (Agilent Technologies, Santa Clara, CA, USA). Quantitative RT-PCR (qRT-PCR) using TaqMan probes and primers (Additional file 12: Table S7) and One-step PCR Master mix (Applied Biosystems, Foster City, CA, USA) was performed with standard curves generated from pooled RNA isolated from the hypothalamus of 10- and 21-day old C57BL/6 J males and females. All the gene expression data were normalized to the level of *PPIB* (Peptidylprolyl Isomerase B = cyclophilin b), the most stable reference gene selected on the basis of NormFinder [75] results.

#### Microarray experiment and differential expression analysis

Total RNA was isolated from hypothalamus of 8 CON and 8 LUN 21-days old F1 females, as described above. RNA with RNA Integrity number ranged 7.9-8.9 (Agilent 2100 Bioanalyzer, Agilent Technologies, Santa Clara, CA) was used for microarray analysis of each individual mouse. Biological replicates were amplified and labeled using the Epicenter TargetAmp Nano-g Biotin-aRNA Labeling Kit for the Illumina system. A total of 750 ng labeled RNA was hybridized to MouseRef-8 v2 BeadChip arrays (Illumina) according to the manufacturer’s protocol. Array data were processed using Illumina GenomeStudio with respect to background subtraction. GeneSpring GX 10 (Agilent Technologies) software was used for further analyses. After array quality control and quantile normalization approx. 16,000 probes were detected. Differentially expressed mRNAs were found by applying fold-change cutoff in either direction at 1.5-fold and threshold *p* < 0.05 corrected with Benjamini-Hochberg false discovery rate. For signaling pathways and molecular functions Ingenuity Pathway Analysis (IPA; http://www.ingenuity.com, accessed April 2015) tool was used. For statistical significance the right-tailed Fisher's exact test using a threshold *p*-value < 0.05 after application of Benjamini-Hochberg method of multiple testing correction was applied. In order to increase clarity of the results: 1) all Canonical Pathways and BioFunctions associated with cancer were removed, and 2) Upstream Analysis involved only genes, RNAs, and proteins. To validate the reliability of the results obtained from the microarray analysis real-time RT-PCR for all genes of interest was performed (Additional file 12: Table S7).

### Statistical analysis

Graphs were created with the GraphPad Prism Software (Version 6.02, GraphPad Software, Inc.; La Jolla, USA). Depending on the experiment data sets were analyzed using two-tailed t test, one-way ANOVA followed by Tukey’s post hoc test or two-way ANOVA followed by Bonferroni’s post hoc test, with age and diet for F1 progeny and age and breeding protocol for F2 progeny as main factors. Linear regression and Pearson correlation coefficients were used to assess associations between body weight and puberty attainment parameters. Differences between the means for all tests were considered statistically significant if *P* < 0.05.

### Data availability

Microarray experiments, described according to MIAME guidelines, have been deposited in the GEO repository (GSE72746).

## Additional files

Additional file 1: Figure S1. Changes in body weight in female (A) and male (B) F1 progeny undernourished (LUN) from birth to weaning at 21 days of age. Body weights were recorded at birth (day 0) by day 55 (for females) or 67 (for males) of age. Asterisks indicate difference between nutritional protocols (*Two-way ANOVA*; ***, *P* < 0.001, ****, *P* < 0.0001). CON – F1 control progeny, LUN – F1 progeny undernourished during lactation. (TIF 1529 kb) Additional file 2: Figure S2. Linear regression analysis establishing association between body weight and age of puberty attainment in F1 progeny undernourished (LUN) from birth to weaning at 21 days of age. (A) Body weight at vaginal opening (VO) and association between body weight and age at VO in female F1 progeny. (B) Body weight at vaginal estrus (VE) and association between body weight and age at VE in female F1 progeny. (C) Body weight at balano preputial separation (BPS) and association between body weight and age at BPS in male F1 progeny. Body weights at VO, VE, and BPS between nutritional protocols were not significant in control (CON) and LUN mice (*t test*; *P* < 0.05). (TIF 1718 kb) Additional file 3: Table S1. Molecular changes in the hypothalami of 10- (D10) and 21-day (D21) old female (A) and male (B) F1 and F2 progeny assessed by real-time RT-PCR. (XLSX 15 kb) Additional file 4: Table S2. List of top 50 genes maintained higher in hypothalami of 21-day old F1 control (CON) vs. undernourished (LUN) females. (DOCX 24 kb) Additional file 5: Table S3. List of top 50 genes maintained lower in hypothalami of 21-day old F1 control (CON) vs. undernourished (LUN) females. (DOCX 23 kb) Additional file 6: Table S4. Top 25 Combined (A) and Single (B) Biofunction Categories enriched with genes showing differential expression in the hypothalamus of control (CON) vs. LUN mice. (XLSX 64 kb) Additional file 7: Table S5. Canonical pathways identified by Ingenuity Pathway Analysis for genes showing differential expression in the hypothalamus of control (CON) vs. LUN mice. Significance for the enrichment of the genes within a particular Canonical pathway was determined by Fisher's exact test right-tailed using the whole database as a reference set (Th = −log(*p*-value) = 1.3). (XLSX 18 kb) Additional file 8: Table S6. Upstream regulators suggested by Ingenuity Pathway Analysis for genes showing differential expression in hypothalamus of control (CON) vs. LUN mice. Ingenuity’s Upstream Regulator Analysis tool predicts upstream regulators from gene expression data based on the literature and compiled in the Ingenuity Knowledge Base. Activation z-score infer the activation state of predicted transcriptional regulator (Th = +/− 1). A Fisher’s exact test *p*-value is calculated to assess the significance of enrichment of the gene expression data for the genes downstream of an upstream regulator. (XLSX 18 kb) Additional file 9: Figure S3. Linear regression analysis establishing association between body weight and age of puberty attainment in female F2 progeny. (A) Body weight at vaginal opening (VO) and association between body weight and age at VO for each breeding protocol (CxC, CxL, LxC, LxL). (B) Body weight at vaginal estrus (VE) and association between body weight and age at VE for each breeding protocol (CxC, CxL, LxC, LxL). Means with different superscripts differ significantly (statistical significance for body weight was calculated by *One-way ANOVA*). C – CON (F1 control progeny), L – LUN (F1 progeny undernourished during lactation). (TIF 1605 kb) Additional file 10: Figure S4. Linear regression analysis establishing associations between body weight and age of puberty attainment in male F2 progeny. Body weight at balano preputial separation (BPS) and association between body weight and age at BPS for each breeding protocol (CxC, CxL, LxC, LxL). Means with different superscripts differ significantly (statistical significance for body weight was calculated by *One-way ANOVA*). C – CON (F1 control progeny), L – LUN (F1 progeny undernourished during lactation). (TIF 1616 kb) Additional file 11: Figure S5. Expression pattern of Kisspeptin-signaling related genes in the hypothalamus of reproductive mature female F2 progeny. Hypothalami were collected in diestrus from 51 ± 0.2-day old females. Expression levels are presented relative to *Ppib* expression (arbitrary units (AU)) for each breeding protocol (CxC, CxL, LxC, LxL). Means with different superscripts differ significantly (statistical significance was calculated by *One-way ANOVA*). C – CON (F1 control progeny), L – LUN (F1 progeny undernourished during lactation). (TIF 1132 kb) Additional file 12: Table S7. Names, primers and probes sequences or assay IDs, RefSeq numbers of genes selected for real-time RT-PCR analysis. (XLSX 13 kb)
