# Supplementary figures and images for: Lactation undernutrition leads to multigenerational molecular programming of hypothalamic gene networks controlling reproduction
Source: BMC Genomics. 2016 May 4;17:333. doi: 10.1186/s12864-016-2615-4 (PMC4857247; doi:10.1186/s12864-016-2615-4)

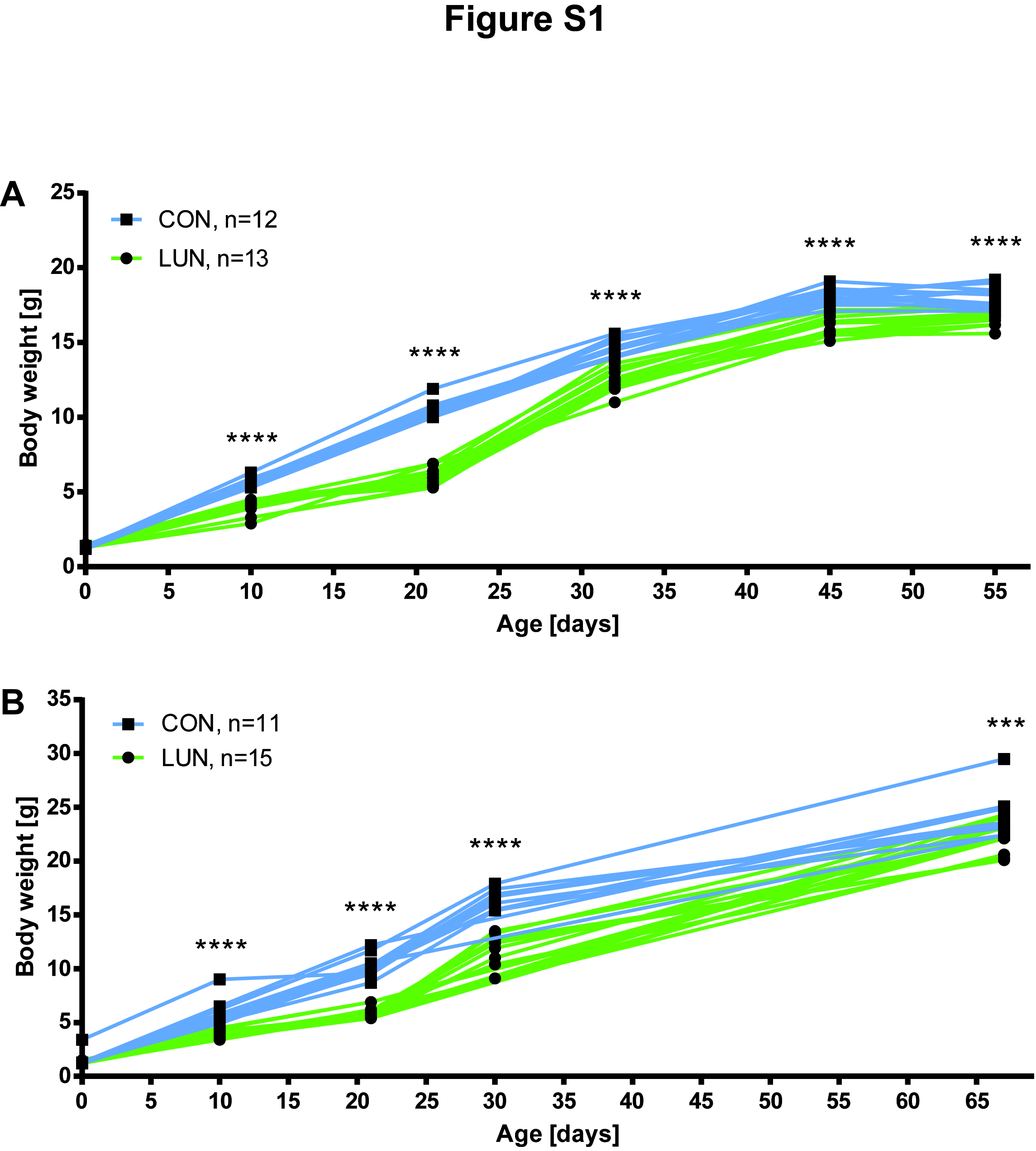

Supplement: Additional file 1: Figure S1. — Changes in body weight in female (A) and male (B) F1 progeny undernourished (LUN) from birth to weaning at 21 days of age. Body weights were recorded at birth (day 0) by day 55 (for females) or 67 (for males) of age. Asterisks indicate difference between nutritional protocols (Two-way ANOVA; ***, P < 0.001, ****, P < 0.0001). CON – F1 control progeny, LUN – F1 progeny undernourished during lactation. (TIF 1529 kb) [file 12864_2016_2615_MOESM1_ESM.tif]

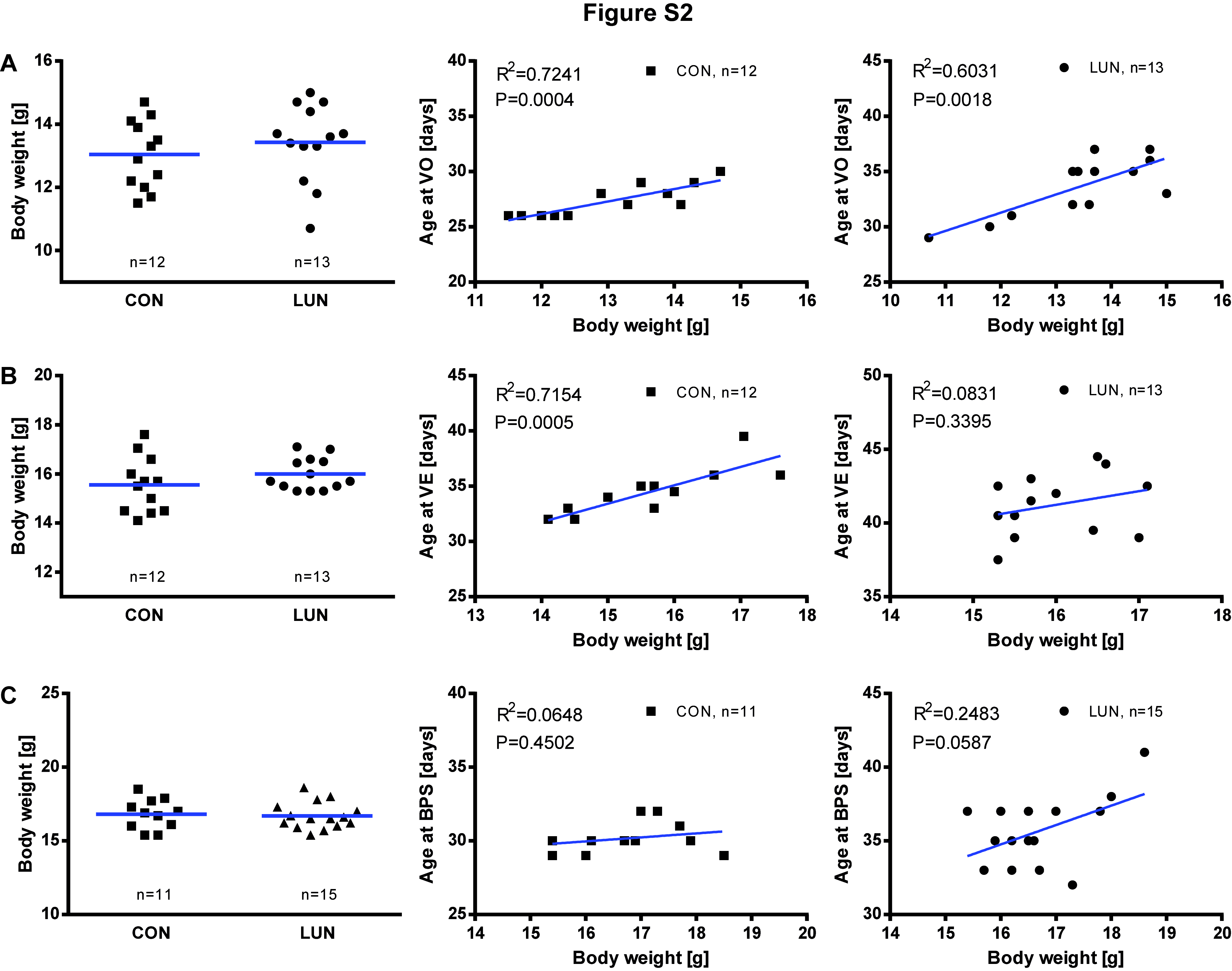

Supplement: Additional file 2: Figure S2. — Linear regression analysis establishing association between body weight and age of puberty attainment in F1 progeny undernourished (LUN) from birth to weaning at 21 days of age. (A) Body weight at vaginal opening (VO) and association between body weight and age at VO in female F1 progeny. (B) Body weight at vaginal estrus (VE) and association between body weight and age at VE in female F1 progeny. (C) Body weight at balano preputial separation (BPS) and association between body weight and age at BPS in male F1 progeny. Body weights at VO, VE, and BPS between nutritional protocols were not significant in control (CON) and LUN mice (t test; P < 0.05). (TIF 1718 kb) [file 12864_2016_2615_MOESM2_ESM.tif]

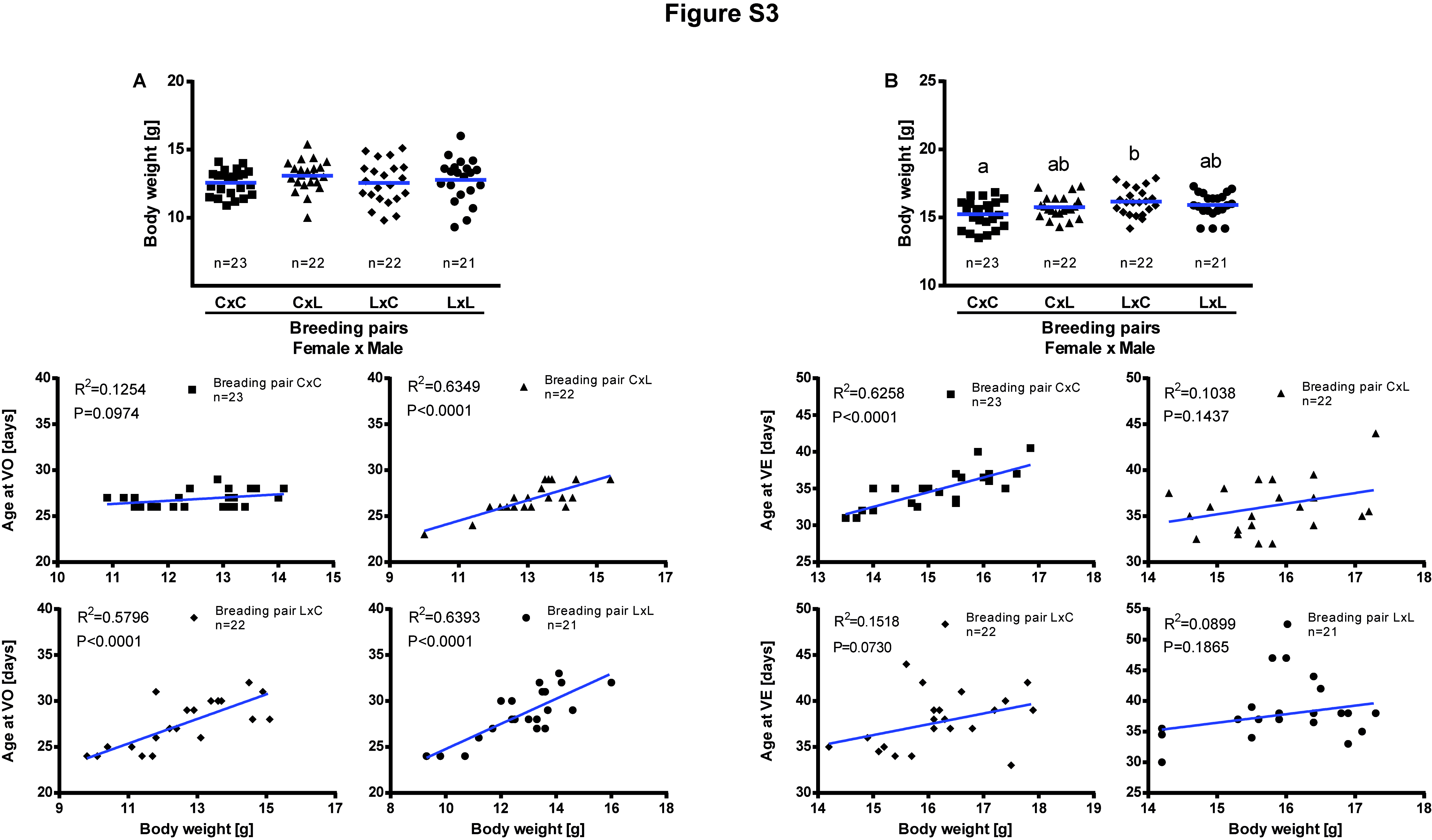

Supplement: Additional file 9: Figure S3. — Linear regression analysis establishing association between body weight and age of puberty attainment in female F2 progeny. (A) Body weight at vaginal opening (VO) and association between body weight and age at VO for each breeding protocol (CxC, CxL, LxC, LxL). (B) Body weight at vaginal estrus (VE) and association between body weight and age at VE for each breeding protocol (CxC, CxL, LxC, LxL). Means with different superscripts differ significantly (statistical significance for body weight was calculated by One-way ANOVA). C – CON (F1 control progeny), L – LUN (F1 progeny undernourished during lactation). (TIF 1605 kb) [file 12864_2016_2615_MOESM9_ESM.tif]

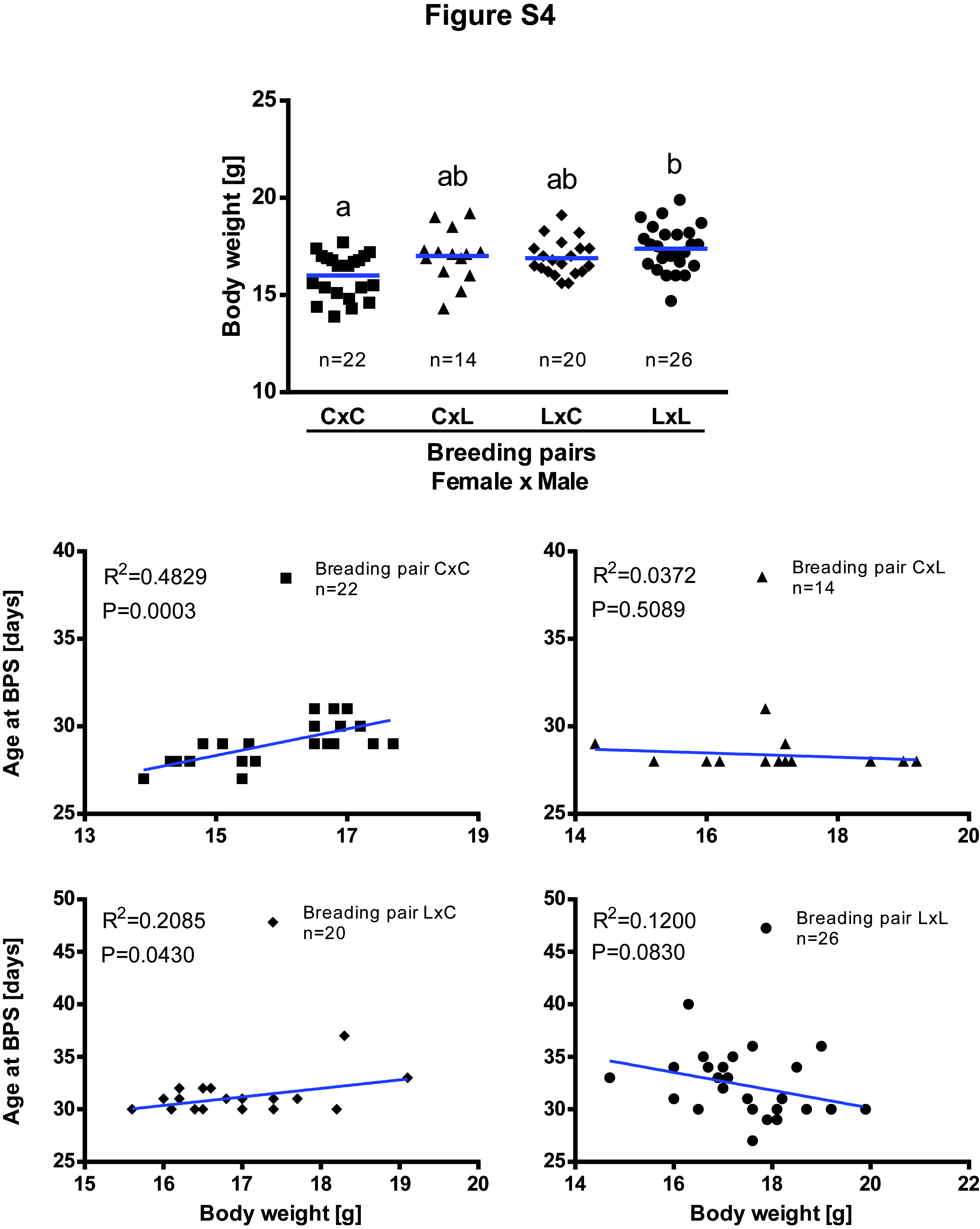

Supplement: Additional file 10: Figure S4. — Linear regression analysis establishing associations between body weight and age of puberty attainment in male F2 progeny. Body weight at balano preputial separation (BPS) and association between body weight and age at BPS for each breeding protocol (CxC, CxL, LxC, LxL). Means with different superscripts differ significantly (statistical significance for body weight was calculated by One-way ANOVA). C – CON (F1 control progeny), L – LUN (F1 progeny undernourished during lactation). (TIF 1616 kb) [file 12864_2016_2615_MOESM10_ESM.tif]

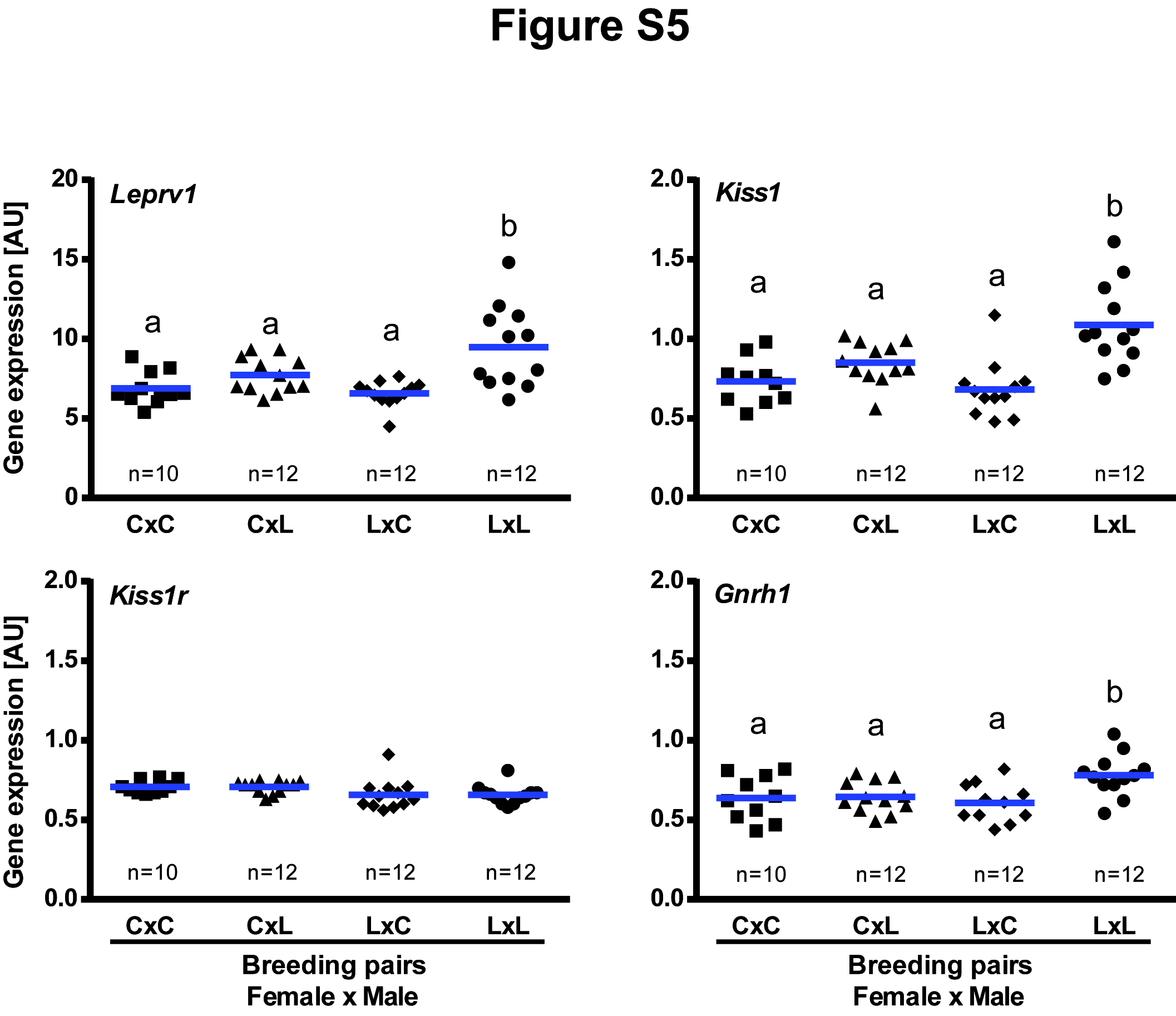

Supplement: Additional file 11: Figure S5. — Expression pattern of Kisspeptin-signaling related genes in the hypothalamus of reproductive mature female F2 progeny. Hypothalami were collected in diestrus from 51 ± 0.2-day old females. Expression levels are presented relative to Ppib expression (arbitrary units (AU)) for each breeding protocol (CxC, CxL, LxC, LxL). Means with different superscripts differ significantly (statistical significance was calculated by One-way ANOVA). C – CON (F1 control progeny), L – LUN (F1 progeny undernourished during lactation). (TIF 1132 kb) [file 12864_2016_2615_MOESM11_ESM.tif]
