# Supplementary material for: Lactation undernutrition leads to multigenerational molecular programming of hypothalamic gene networks controlling reproduction
Source: BMC Genomics. 2016 May 4;17:333. doi: 10.1186/s12864-016-2615-4 (PMC4857247; doi:10.1186/s12864-016-2615-4)
Supplement: Additional file 4: Table S2. — List of top 50 genes maintained higher in hypothalami of 21-day old F1 control (CON) vs. undernourished (LUN) females. (DOCX 24 kb) [file 12864_2016_2615_MOESM4_ESM.docx]

**Table S2. List of top 50 genes maintained higher in hypothalami of 21-day old F1 control (CON) *vs.* undernourished (LUN) females.**

| **Symbol** | **Entrez ID** | **Entrez Gene Name** | **Fold change** |
| --- | --- | --- | --- |
| **POMC** | 18976 | proopiomelanocortin | 9.950 |
| **Gypa** | 14934 | glycophorin A | 7.404 |
| **TSGA10** | 211484 | testis specific. 10 | 6.400 |
| **KISS1** | 280287 | KiSS-1 metastasis-suppressor | 5.225 |
| **Ahsp** | 170812 | alpha hemoglobin stabilizing protein | 4.877 |
| **TSPAN8** | 216350 | tetraspanin 8 | 4.020 |
| **SDCCAG3** | 68112 | serologically defined colon cancer antigen 3 | 3.603 |
| **NETO1** | 246317 | neuropilin (NRP) and tolloid (TLL)-like 1 | 3.164 |
| **NKTR** | 18087 | natural killer-tumor recognition sequence | 3.111 |
| **SLC4A1** | 20533 | solute carrier family 4. anion exchanger. member 1 | 3.108 |
| **ZNF329** | 67230 | zinc finger protein 329 | 3.011 |
| **AI504432** | 229694 | expressed sequence AI504432 | 3.002 |
| **GABPB2** | 213054 | GA binding protein transcription factor. beta subunit 2 | 2.947 |
| **XPO1** | 103573 | exportin 1 (CRM1 homolog. yeast) | 2.941 |
| **MARC2** | 67247 | mitochondrial amidoxime reducing component 2 | 2.906 |
| **GATAD2B** | 229542 | GATA zinc finger domain containing 2B | 2.893 |
| **MRPL15** | 27395 | mitochondrial ribosomal protein L15 | 2.619 |
| **CCP110** | 101565 | centriolar coiled coil protein 110kDa | 2.555 |
| **COMMD2** | 52245 | COMM domain containing 2 | 2.546 |
| **RNASE1** | 19752 | ribonuclease. RNase A family. 1 (pancreatic) | 2.530 |
| **ENTPD3** | 215446 | ectonucleoside triphosphate diphosphohydrolase 3 | 2.510 |
| **PKD2L1** | 329064 | polycystic kidney disease 2-like 1 | 2.453 |
| **GTF2A1** | 83602 | general transcription factor IIA. 1. 19/37kDa | 2.426 |
| **PIK3R1** | 18708 | phosphoinositide-3-kinase. regulatory subunit 1 (alpha) | 2.411 |
| **Cbx3** | 12417 | chromobox 3 | 2.349 |
| **SPAG9** | 70834 | sperm associated antigen 9 | 2.305 |
| **ZFP106** | 20402 | zinc finger protein 106 homolog (mouse) | 2.295 |
| **SLC43A2** | 215113 | solute carrier family 43. member 2 | 2.276 |
| **LRRC39** | 109245 | leucine rich repeat containing 39 | 2.258 |
| **CDC5L** | 71702 | cell division cycle 5-like | 2.247 |
| **HBA1/HBA2** | 15122 | hemoglobin. alpha 1 | 2.227 |
| **SLC7A14** | 241919 | solute carrier family 7 (orphan transporter). member 14 | 2.210 |
| **Lyz1/Lyz2** | 17105 | lysozyme 2 | 2.171 |
| **Hist1h4m*** | 319161 | histone cluster 1. H4m | 2.165 |
| **PALM2** | 242481 | paralemmin 2 | 2.144 |
| **Ccdc162** | 75973 | coiled-coil domain containing 162 | 2.135 |
| **GNGT2** | 14710 | guanine nucleotide binding protein (G protein). γ transducing activity polypeptide 2 | 2.119 |
| **FBXL21** | 213311 | F-box and leucine-rich repeat protein 21 (gene/pseudogene) | 2.118 |
| **SERPIND1** | 15160 | serpin peptidase inhibitor. clade D (heparin cofactor). member 1 | 2.114 |
| **MCM2** | 17216 | minichromosome maintenance complex component 2 | 2.091 |
| **FBLN1** | 14114 | fibulin 1 | 2.088 |
| **C8orf4** | 69068 | chromosome 8 open reading frame 4 | 2.080 |
| **SCGN** | 214189 | secretagogin. EF-hand calcium binding protein | 2.073 |
| **MFAP4** | 76293 | microfibrillar-associated protein 4 | 2.041 |
| **DUSP18** | 75219 | dual specificity phosphatase 18 | 2.040 |
| **LOC100046406** | 100046406 |  | 2.033 |
| **PCOLCE** | 18542 | procollagen C-endopeptidase enhancer | 2.006 |
| **NUSAP1** | 108907 | nucleolar and spindle associated protein 1 | 2.003 |
| **CTNNBL1** | 66642 | catenin. beta like 1 | 1.967 |
| **MPP5** | 56217 | membrane protein. palmitoylated 5 (MAGUK p55 subfamily member 5) | 1.955 |

*Categorized to Hist2h4 family in IPA.
