# Supplementary material for: Lactation undernutrition leads to multigenerational molecular programming of hypothalamic gene networks controlling reproduction
Source: BMC Genomics. 2016 May 4;17:333. doi: 10.1186/s12864-016-2615-4 (PMC4857247; doi:10.1186/s12864-016-2615-4)
Supplement: Additional file 5: Table S3. — List of top 50 genes maintained lower in hypothalami of 21-day old F1 control (CON) vs. undernourished (LUN) females. (DOCX 23 kb) [file 12864_2016_2615_MOESM5_ESM.docx]

**Table S3. List of top 50 genes maintained lower in hypothalami of 21-day old F1 control (CON) *vs.* undernourished (LUN) females.**

| **Symbol** | **Entrez ID** | **Entrez Gene Name** | **Fold change** |
| --- | --- | --- | --- |
| ENDOU | 19011 | endonuclease, polyU-specific | -10.697 |
| SHOX2 | 20429 | short stature homeobox 2 | -9.968 |
| KLK1* | 16612 | kallikrein-related peptidase 1 | -9.607 |
| VIL1 | 22349 | villin 1 | -8.655 |
| SYNPO2 | 118449 | synaptopodin 2 | -8.622 |
| MYL2 | 17906 | myosin, light chain 2, regulatory, cardiac, slow | -7.208 |
| SLC17A7 | 72961 | solute carrier family 17 (sodium-dependent inorganic phosphate cotransporter), member 7 | -5.998 |
| EPHA1 | 13835 | EPH receptor A1 | -4.968 |
| GRID2IP | 170935 | glutamate receptor, ionotropic, delta 2 (Grid2) interacting protein | -4.613 |
| USP43 | 216835 | ubiquitin specific peptidase 43 | -4.602 |
| SRPX2 | 68792 | sushi-repeat containing protein, X-linked 2 | -4.393 |
| KLK1b5* | 16622 | kallikrein 1-related peptidase b5 | -4.150 |
| RAB38 | 72433 | RAB38, member RAS oncogene family | -3.617 |
| WNT9B | 22412 | wingless-type MMTV integration site family, member 9B | -3.587 |
| PRKCD | 18753 | protein kinase C, delta | -3.574 |
| TNNT1 | 21955 | troponin T type 1 (skeletal, slow) | -3.566 |
| FZD10 | 93897 | frizzled family receptor 10 | -3.389 |
| RASD1 | 19416 | RAS, dexamethasone-induced 1 | -3.347 |
| BOK | 51800 | BCL2-related ovarian killer | -3.325 |
| RAMP3 | 56089 | receptor (G protein-coupled) activity modifying protein 3 | -3.310 |
| KRT12 | 268482 | keratin 12 | -3.200 |
| GABRD | 14403 | gamma-aminobutyric acid (GABA) A receptor, delta | -3.144 |
| ATP2A1 | 11937 | ATPase, Ca++ transporting, cardiac muscle, fast twitch 1 | -3.133 |
| LEF1 | 16842 | lymphoid enhancer-binding factor 1 | -3.125 |
| NTNG1 | 80883 | netrin G1 | -2.996 |
| Wfdc10 | 629756 | WAP four-disulfide core domain 10 | -2.971 |
| CWH43 | 231293 | cell wall biogenesis 43 C-terminal homolog (S. cerevisiae) | -2.966 |
| GBX2 | 14472 | gastrulation brain homeobox 2 | -2.953 |
| NKX3-1 | 18095 | NK3 homeobox 1 | -2.886 |
| SLC12A8 | 171286 | solute carrier family 12 (potassium/chloride transporters), member 8 | -2.882 |
| SCUBE2 | 56788 | signal peptide, CUB domain, EGF-like 2 | -2.777 |
| SCEL | 64929 | sciellin | -2.633 |
| ADRA2B | 11552 | adrenoceptor alpha 2B | -2.588 |
| CALCA^ | 12310 | calcitonin-related polypeptide alpha | -2.572 |
| AGAP2 | 216439 | ArfGAP with GTPase domain, ankyrin repeat and PH domain 2 | -2.493 |
| PRKCH | 18755 | protein kinase C, eta | -2.459 |
| TCF7L2 | 21416 | transcription factor 7-like 2 (T-cell specific, HMG-box) | -2.441 |
| COL17A1 | 12821 | collagen, type XVII, alpha 1 | -2.396 |
| TRPM6 | 225997 | transient receptor potential cation channel, subfamily M, member 6 | -2.358 |
| FOXP2 | 114142 | forkhead box P2 | -2.346 |
| NMBR | 18101 | neuromedin B receptor | -2.346 |
| CCK | 12424 | cholecystokinin | -2.334 |
| EPS8L2 | 98845 | EPS8-like 2 | -2.333 |
| STK32C | 57740 | serine/threonine kinase 32C | -2.290 |
| SLITRK6 | 239250 | SLIT and NTRK-like family, member 6 | -2.237 |
| IYD | 70337 | iodotyrosine deiodinase | -2.235 |
| DCTN1 | 13191 | dynactin 1 | -2.231 |
| FAM19A4 | 320701 | family with sequence similarity 19 (chemokine (C-C motif)-like), member A4 | -2.230 |
| OTOA | 246190 | otoancorin | -2.223 |
| MAGEB1^#^ | 65956 | melanoma antigen, family B, 1 | -2.196 |

*Categorized to KLK3 family in IPA. ^#^Categorized to CCL21 family in IPA. ^ Categorized to CALCB family in IPA.
